# HSP60 critically regulates endogenous IL-1β production in activated microglia by stimulating NLRP3 inflammasome pathway

**DOI:** 10.1186/s12974-018-1214-5

**Published:** 2018-06-09

**Authors:** Shalini Swaroop, Anita Mahadevan, Susarla Krishna Shankar, Yogita K. Adlakha, Anirban Basu

**Affiliations:** 1 National Brain Research Centre, Manesar, Haryana 122052 India; 2 Department of Neuropathology, National Institute of Mental Health and Neurosciences, Bangalore, India

**Keywords:** Microglia, IL-1β, Heat shock protein, HSP60, Inflammation, Mitochondrial stress, NLRP3, Inflammasome, Japanese encephalitis virus (JEV), ROS, Caspase-1

## Abstract

**Background:**

Interleukin-1β (IL-1β) is one of the most important cytokine secreted by activated microglia as it orchestrates the vicious cycle of inflammation by inducing the expression of various other pro-inflammatory cytokines along with its own production. Microglia-mediated IL-1β production is a tightly regulated mechanism which involves the activation of nucleotide-binding oligomerization domain leucine-rich repeat and pyrin domain-containing 3 (NLRP3) inflammasome pathway. Our previous study suggests the critical role of heat shock protein 60 (HSP60) in IL-1β-induced inflammation in microglia through TLR4-p38 MAPK axis. However, whether HSP60 regulates endogenous IL-1β production is not known. Therefore, to probe the underlying mechanism, we elucidate the role of HSP60 in endogenous IL-1β production.

**Methods:**

We used in vitro (N9 murine microglial cells) and in vivo (BALB/c mouse) models for our study. HSP60 overexpression and knockdown experiment was done to elucidate the role of HSP60 in endogenous IL-1β production by microglia. Western blotting and quantitative real-time PCR was performed using N9 cells and BALB/c mice brain, to analyze various proteins and transcript levels. Reactive oxygen species levels and mitochondrial membrane depolarization in N9 cells were analyzed by flow cytometry. We also performed caspase-1 activity assay and enzyme-linked immunosorbent assay to assess caspase-1 activity and IL-1β production, respectively.

**Results:**

HSP60 induces the phosphorylation and nuclear localization of NF-κB both in vitro and in vivo. It also induces perturbation in mitochondrial membrane potential and enhances reactive oxygen species (ROS) generation in microglia. HSP60 further activates NLRP3 inflammasome by elevating NLRP3 expression both at RNA and protein levels. Furthermore, HSP60 enhances caspase-1 activity and increases IL-1β secretion by microglia. Knockdown of HSP60 reduces the IL-1β-induced production of IL-1β both in vitro and in vivo. Also, we have shown for the first time that knockdown of HSP60 leads to decreased IL-1β production during Japanese encephalitis virus (JEV) infection, which eventually leads to decreased inflammation and increased survival of JEV-infected mice.

**Conclusion:**

HSP60 mediates microglial IL-1β production by regulating NLRP3 inflammasome pathway and reduction of HSP60 leads to reduction of inflammation in JEV infection.

**Electronic supplementary material:**

The online version of this article (10.1186/s12974-018-1214-5) contains supplementary material, which is available to authorized users.

## Background

Neuroinflammation being the first line of defense of the central nervous system (CNS) is a complex biochemical process by which brain and spinal cord react to diverse pathogenic and harmful stimuli including host-derived danger signal of cellular damage [1]. However, uncontrolled neuroinflammation may lead to tissue injury and neuronal death and has been identified as a causative factor of multiple neurological diseases [2–4]. Microglia, the defense cells of the CNS, play a major role in neuroinflammation [5]. They get activated by sensing harmful stimuli such as pathogenic invasion, neuronal damage, and neurodegeneration which results in the upregulation of various pro- and anti-inflammatory factors (such as interleukin-1β (IL-1β), tumor necrosis factor-α (TNF-α), monocyte chemoattractant protein-1 (MCP-1), interleukin-6 (IL-6), interleukin-10 (IL-10), inducible nitric oxide synthase (iNOS), cyclooxygenase-2 (COX2)) to combat neuronal damage [3]. However, overactivation of microglia can cause excess production of pro-inflammatory factors, reactive oxygen species (ROS), and nitric oxide (NO) and can result in neuroinflammation, oxidative stress, and neurodegeneration.

Interleukin-1β is a potent pro-inflammatory cytokine which gets secreted by activated microglia and plays a crucial role in neuroinflammation and constitutive activation of microglia, and therefore, it is considered as the master regulator of inflammation [6–9]. It is a multifunctional protein and is able to induce the expression of other pro-inflammatory factors besides its own secretion and thus starts a vicious cycle of inflammation and forms the feed-forward loop of inflammatory response [7, 10–13]. Secretion of IL-1β also involves the activation of inflammasome complex, a subcellular multiprotein complex that assembles in the cytosol after sensing a wide range of pathogen-associated molecular patterns (PAMPs) and damage-associated molecular patterns (DAMPs) [14–17]. Assembly of inflammasome complex, in turn, triggers proteolytic cleavage of pro-caspase-1 into active caspase-1 and helps in the maturation of IL-1β from its precursor form, thus inducing inflammation and pyroptosis [18]. Inflammasome complex mainly comprises three domains—(i) pattern recognition receptors (PRRs), (ii) the adaptor, mostly an apoptosis-associated spec-like protein containing a caspase-recruitment domain (ASC-CARD), and (iii) the caspase (cysteine protease). Mainly five types of PRRs (NLRP1, NLRP3, NLRC3, pyrin, and AIM2) have been reported to contribute in inflammasome complex [19]. Among these, nucleotide-binding oligomerization domain leucine-rich repeat and pyrin domain-containing 3 (NLRP3) has been shown to have a promising role in neuroinflammation [20]. It can sense various stimuli and forms a molecular platform for caspase-1 activation, which leads to the processing and release of IL-1β and IL-18, thus eventually potentiating inflammatory responses that are involved in multiple infections, inflammatory, and immune diseases [15, 21]. Thus, the NLRP3 inflammasome is of crucial importance in the development of both acute and chronic inflammatory responses.

Despite this extensive knowledge, the detailed mechanism of endogenous IL-1β production in activated microglia is not well understood. The biggest challenge of developing anti-neuroinflammatory therapy for various neurodegenerative diseases in the field of neuroinflammation necessitated this study. In our previous study, we performed proteomic analysis of IL-1β-treated N9 murine microglial cells to identify the differentially expressed proteins involved in microglial activation and neuroinflammation. We discovered that HSP60, an important mitochondrial chaperone protein, which gets upregulated in microglia in response to IL-1β treatment, acts as a key hub molecule. We also established that HSP60 regulates IL-1β-induced inflammation in microglia via a TLR4-p38 MAPK axis [10]. Several other studies have shown the immunomodulatory role of HSP60 during pathogenic invasion and as a neuroglia crosstalk molecule during neurodegeneration [22, 23]. Reports also suggest that HSP60 acts as a link between mitochondrial stress and inflammation and stimulates cytokine production [24, 25]. However, our understanding about the crucial role of HSP60 in the endogenous IL-1β production remains limited due to the absence of in vivo evidence. Hence, extending our previous study [10], we set out to unravel the role of HSP60 in IL-1β-induced endogenous IL-1β production using in vitro and in vivo model. Here, we demonstrate that HSP60 enhances mitochondrial stress and activates NLRP3 inflammasome complex during IL-1β production.

As we observed a significant role of HSP60 in IL-1β production by activated microglia, this prompted us to explore its role in a disease condition where inflammation has a distinct role in guiding the pathology. Japanese encephalitis virus (JEV), a positive single-stranded virus, causes such a severe brain pathology and also initiates a potent inflammatory response, due to which about one third of the patients die after JEV infection and half of the survivors suffer permanent neuropsychiatric sequelae [26, 27]. It causes robust microglial activation, increase in IL-1β production, and inflammation that enhances the severity of infection [28–30]. Literature suggests that JEV induces IL-1β production by stimulating NLRP3 inflammasome complex activation in microglia [29, 30] and resultant increased inflammation leads to the bystander death of the animal. In our lab, we have a well-established model of JEV infection which is a relevant model to study neuroinflammation; therefore, we were curious to know whether modulation of HSP60 can ameliorate IL-1β production and subsequent inflammation caused by JEV infection. Here, for the first time, we show that attenuating HSP60 expression in JEV-infected mice leads to robust decline in IL-1β production and thus ameliorates JEV-induced inflammation which, in turn, leads to enhanced survival.

## Methods

### Animal experiments

All the animal experiments were performed after obtaining approval from the Institutional Animal Ethics Committee of the National Brain Research Centre (NBRC) (NBRC/IAEC/2016/115 and NBRC/IAEC/2017/028). For in vivo experiments, postnatal day 8–10 (P08-P10) BALB/c mice were used, irrespective of their sex. The animals were handled in strict accordance with good animal practice as per the guidelines of the Committee for the Purpose of Control and Supervision of Experiments on Animals, Ministry of Environment and Forestry, Government of India.

### IL-1β and morpholino treatment in mice

IL-1β was injected intraperitoneally (i.p.) at a dose of 10 ng/g of body weight of P10 BALB/c mice pups after every 24 h for different durations (1, 2, and 3 days) as described earlier [13]. Control mice group received intraperitoneal injection of equal volume of PBS.

Vivo-morpholino are morpholino oligos coupled with eight guanidinium head groups on dendrimer scaffold that enable delivery into cells [31]. Morpholino oligomers are proven antisense molecules used for the specific knockdown of the gene of interest both in vitro and in vivo*.* It either blocks the mRNA translation or interferes with RNA processing, including splicing and mRNA maturation [32]. HSP60 vivo-morpholino (HSP60-Mo) oligos were commercially procured from Gene Tools LLC (Philomath, OR, USA). HSP60-Mo was designed against sequences of mouse HSP60 (HSPD1) gene to specifically target it (5′ ACT GTG GGT AGT CGA TTT CT 3′). A 25-base scrambled morpholino of random sequence (SC-Mo) was used as a negative control (5′ TGG TTT CAG AAT AGT ATT CCA CTG C 3′).

For in vivo IL-1β experiments, animals were divided into six groups: (i) Control, (ii) IL-1β treatment, (iii) Sc-Mo, (iv) Sc-Mo + IL-1β treatment, (v) HSP60-Mo, and (vi) HSP60-Mo + IL-1β treatment group. Each group had a minimum of three animals. Among these, groups (v) and (vi) were intracranially injected with HSP60 vivo-morpholino at P8 (15 mg/kg of body weight of mice), while groups (iii) and (iv) received intracranial injection of scrambled vivo-morpholino at P8 (15 mg/kg of body weight of mice). As efficiency of vivo-morpholino in crossing the blood brain barrier is quite low, therefore, to achieve significant knockdown in the brain, our laboratory devised a slightly different strategy based on a previously published method [33, 34]. The intracranial injection was given manually in 8-day-old BALB/c mice pups (P8) at a single site as vivo-morpholino is believed to diffuse in the tissue [35, 36]. The amount of vivo-morpholino was calculated according to the body weight of each mice and calculated volume of vivo-morpholino was made up to 25 μl using 1× PBS. Then this 25 μl of vivo-morpholino solution was taken in the insulin syringe with needle size 31 G × 15/64 (0.25 × 6 mm), and it was slowly injected by gently pushing the syringe piston. Groups (i) and (ii) received an intracranial injection of PBS at P8 (same volume as vivo-morpholino). At P10, IL-1β was injected intraperitoneally (i.p.) in groups (ii), (iv), and (vi) at a dose of 10 ng/g of body weight of mice pups dissolved in 50 μl PBS, for three consecutive days. Groups (i), (iii), and (v) received same volume of PBS intraperitoneally. The mice were then sacrificed by repeated transcardial perfusion of chilled PBS and their brains were collected for protein and/or RNA analysis. The efficacy of the morpholino injection and its efficiency to knockdown HSP60 was checked through Western blot which was done by random sampling for morpholino-treated group. After we observed specific knockdown of HSP60 by vivo-morpholino (Additional file 1: Figure S1(A)), then only we proceeded for further experiments using vivo-morpholino with following four groups: (i) Control, (ii) IL-1β, (iii) HSP60-Mo, and (iv) HSP60-Mo + IL-1β groups.

### Cell culture, IL-1β treatment, and transfections

All the in vitro experiments were performed in N9 murine microglial cells (N9 cells), which were a kind gift from Prof. Maria Pedroso de Lima (Center for Neuroscience and Cell Biology, University of Coimbra, Portugal) and were grown as described earlier [10]. N9 cells were chosen for the study as these microglial cells were derived from mouse brains and share many phenotypic characteristics with primary mouse microglia [37]. Transfection of HSP60 plasmid and endonuclease-prepared short interfering RNA (esiRNA) against mouse HSP60 gene was performed in N9 cells as described earlier for overexpression and knockdown experiments [10]. For the overexpression studies, 4 μg recombinant mouse HSP60 plasmid (MC206740, Origene) was used (Additional file 1: Figure S2), while 5 pM HSP60 eSiRNA (EMU151751, Sigma Aldrich) was used for knockdown experiments.

To induce inflammation, the N9 cells were serum starved for 2 h at 70% confluency and treated with 5 ng/ml IL-1β for different time periods. The cells were then used for different assays. For Western blotting, caspase-1 assay, and enzyme-linked immunosorbent assay, 1.5 × 10^6^ cells were seeded in 90 mm × 20 mm plates, while for quantitative real-time PCR and flow cytometric analysis (reactive oxygen species analysis, cytokine bead array, and rhodamine 123 assays), 6 × 10^5^ cells were seeded in 60 mm × 15 mm plates.

### JEV infection of mice and N9 cells

Viral suspensions were prepared from the mice brain using the GP78 strain of JEV as described previously [38]. P10 BALB/c mouse pups were divided into six groups: (i) Control, (ii) JEV- infected, (iii) only Sc-Mo, (iv) Sc-Mo + JEV, (v) only HSP60-Mo, and (vi) HSP60-Mo + JEV group, and each group had a minimum of three pups. The HSP60-Mo group and HSP60-Mo + JEV infection group received an intracranial injection of HSP60-Mo at P8 (15 mg/kg of body weight of mice), while Sc-Mo and Sc-Mo + JEV groups were intracranially injected with scrambled vivo-morpholino (15 mg/kg of body weight of mice). Control and only JEV-infected groups received intracranial injection of PBS (same volume as vivo-morpholino) at P8. Mice from JEV group, Sc-Mo + JEV group, and HSP60-Mo + JEV group were injected with 1.5 × 10^3^ plaque-forming units (PFU) of virus in 50 μl PBS, whereas control group, Sc-Mo group, and HSP60-Mo group were given same volume of PBS, intraperitoneally. After the development of full symptoms (including tremors, ruffled fur, hunching, ataxia, gait abnormalities like hind limb paralysis and body stiffening), the animals were sacrificed and their brains were excised after repeated transcardial perfusion with ice-cold PBS. The animal brains were then used for either protein or RNA analysis. Knockdown of HSP60 by vivo-morpholino was confirmed at protein levels by Western blotting (Additional file 1: Figure S1(B)). After confirming specific knockdown of HSP60 in JEV-infected group by HSP60 Mo, we proceeded with following four groups for further experiments: (i) Control, (ii) JEV-infected, (iii) only HSP60-Mo, and (iv) HSP60-Mo + JEV group.

For the JEV infection of N9 cells, around 1.5 × 10^6^ cells were seeded in 90 mm × 20 mm plates in 5% RPMI and were allowed to grow for 12–15 h. After the cells reached 70% confluence, they were serum starved for 2 h and infected with JEV (strain GP78) at an MOI (multiplicity of infection) of 2 followed by incubation at 37 °C for 24 h to induce inflammation. The MOI of 2 was chosen for JEV infection as it significantly induces inflammation as compared to low MOI (Additional file 1: Figure S3). The cells were then harvested to isolate RNA for quantitative real-time PCR and protein for cytokine bead array and Western blotting.

### Human brain tissues

Fresh frozen paraffin-embedded (FFPE) human brain tissue sections were obtained from the Human Brain Tissue Repository, National Institute of Mental Health and Neurosciences, Bangalore, India, in accordance with the institutional scientific ethics, protecting the confidentiality of the subjects. These sections were obtained from the frontal cortex/hippocampus postmortem from at least two confirmed patients of different brain disorders. For the control experimental sets, brain tissues from individuals who succumbed to traffic accidents and had no known prior neurological disease were used. The human glioma brain tissues were kindly provided by Dr. Ellora Sen (NBRC).

### RNA isolation and quantitative real-time PCR (qRT-PCR) from tissues and cells

The 5-μm-thick FFPE frontal cortex sections were deparaffinized by repeated incubation in xylene followed by washing in alcohol gradient. Age-matched control samples were obtained from accidental cases with least possible trauma to brain. The RNA isolation was performed from human FFPE sections, human glioma brain tissue, N9 cells, and mouse brains using Tri reagent (Sigma-Aldrich), and cDNA was synthesized using an Advantage RT-for-PCR kit (Clontech Laboratories) as per manufacturer’s protocol. qRT-PCR was carried out as described previously [10] from 500 ng RNA, using primers specific for mouse IL-1β, HSP60, and NLRP3 genes. The conditions used for the qRT-PCR were as follows: 95 °C for 3 min (1 cycle) and 94 °C for 20 s, 55 °C for 30 s, and 72 °C for 45 s (40 cycles). The relative mRNA abundance was determined by normalizing to GAPDH mRNA using the Pfaffl method [39]. To elucidate the changes in IL-1β and HSP60 transcript levels in different brain disorders, two different qRT-PCRs were carried out (for IL-1β and HSP60) for each neurological condition. The qRT-PCR was done in triplicates. The primer sequences used for qRT-PCR analysis are listed in Additional file 1: Table S1.

### Protein isolation

#### Cytosolic protein isolation

##### From N9 cells

Cytosolic protein fractions from N9 cells were isolated as described earlier [10, 13]. Briefly, around 3 × 10^6^ were pelleted down and lysed in 100 μl lysis buffer containing 1% Triton-X-100, 10 mM Tris (hydroxymethyl) aminomethane-Cl (pH 8.0), 0.2% ethylene glycol tetraacetic acid, 1 mM ethylenediaminetetraacetic acid, 150 mM sodium chloride, 0.5% octylphenoxypolyethoxyethanol (Nonidet P-40), 0.2% sodium orthovanadate, and protease inhibitor cocktail (Sigma Aldrich). The samples were sonicated and the lysates were centrifuged at 12,000*g* for 30 min at 4 °C, followed by collection of supernatant containing cytosolic protein fraction. The protein was quantified using bicinconinic acid (BCA) method.

##### From BALB/c mice brains

For the cytosolic protein isolation from the brain samples, mice brain tissue was homogenized in 500 μl lysis buffer (composition mentioned above) to obtain cell suspension. The lysate was then sonicated and centrifuged at 12,000*g* for 30 min at 4 °C and supernatant was collected.

#### Nuclear protein isolation

##### From N9 cells

For the nuclear protein isolation, the untreated and treated cells were first lysed in buffer A (containing 10 mM HEPES (4-(2-hydroxyethyl)-1-piperazineethanesulfonic acid, 10 mM KCl, 0.1 mM ethylenediaminetetraacetic acid (EDTA), 0.1 mM ethylene glycol-bis (β-aminoethyl ether)-N,N,N′,N′-tetraacetic acid (EGTA), 1 mM dithiothreitol (DTT), 0.5 mM phenylmethylsulfonyl fluoride (PMSF), nonionic surfactant, octylphenoxypolyethoxyethanol (IGEPAL), 0.2% sodium orthovanadate (SOV), and protease inhibitor cocktail (PIC) (Sigma Aldrich) for 30 min followed by centrifugation at 14,000*g* at 4 °C for 5 min. After discarding the supernatant**,** pellet was resuspended and sonicated in ice-cold buffer B containing 20 mM HEPES, 100 mM KCl, 1 mM EDTA, 0.2% SOV, and PIC. The lysate was centrifuged at 15,000*g* at 4 °C for 20 min. The nuclear extract was collected as supernatant and was estimated using BCA method.

##### From BALB/c mice brains

For the nuclear protein isolation from BALB/c mice brains, the whole brain tissues were first homogenized in buffer A (composition mentioned above) and the cell suspension was obtained. After this, the same protocol was followed to obtain nuclear protein from the brain cell suspension as used for in vitro N9 cell culture. The nuclear protein was then quantified by BCA method and was used for Western blotting.

### Western blotting

Western blotting was performed as previously described [10]. Around 3 × 10^6^ cells were pelleted and protein was isolated and quantified by the abovementioned protocol. For Western blotting of cytosolic as well as nuclear protein fractions, 30 μg protein was used. Primary antibodies against the following proteins were used: HSP60 (Abcam, #Ab46798), NLRP3 (Abcam, #Ab91525), inducible nitric oxide synthase (iNOS) (Abcam, #Ab3523), phospho-p65 nuclear factor-κB (NF-κB) p65 (S536) (Cell signaling Technology, #3033), Proliferating cell nuclear antigen (PCNA) (Cell Signaling Technology, #2586), Cycloxygenase-2 (COX2) (Millipore, #Ab5118), NF-κBp-65 (Santa Cruz Biotechnology, #SC372), and β-actin (Sigma Aldrich, #A3854). Secondary antibodies were labeled with horseradish peroxidase. The images were captured and analyzed using the Uvitec gel documentation system (Cambridge, UK) and ImageJ software respectively. Cytosolic protein levels were normalized to β-actin levels, whereas nuclear protein levels were normalized to PCNA. The fold change with respect to control cells was then calculated based on integrated density values (IDV).

### Cytokine bead array (CBA)

For the quantitative analysis of various important cyto-chemokines in untreated and treated cells, the CBA kit (BD Biosciences, NJ, USA) was used. Around 1.5 × 10^6^ cells were pelleted down and protein was isolated and quantified. The beads coated with antibodies against interleukin 6 (IL-6), tumor necrosis factor alpha (TNF-α), and monocyte chemoattractant protein-1 (MCP-1) antibodies were mixed with 50 μg cell lysates and standards according to the manufacturer’s instructions. The experiments were performed in triplicates as described earlier [13]. A total of 10,000 events were acquired for each sample. The results were analyzed using FACS Calibur (Becton Dickinson) and CBA software that allows the calculation of cytokine concentrations in unknown lysates with the help of a standard curve.

### Reactive oxygen species (ROS) measurement

The levels of ROS generated within N9 cells of each treatment groups were measured by the CM-H2DCFDA (5 (and 6)-chlromethyl-20,70-dichloro-dihydrofluoresceindiacetate) (Sigma Aldrich), which is a cell-permeable, non-polar, H_2_O_2_-sensitive probe. It diffuses into cells, where intracellular esterases cleave its acetate groups, releasing the corresponding dichlorodihydrofluorescein derivative which gives red fluorescence [30]. 6 × 10^5^ cells were seeded for the ROS analysis. After the completion of treatment, the untreated and treated N9 cells were incubated with 5 μM CM-H2DCFDA in dark at 37 °C room temperature for 20 min followed by washing and the relative mean fluorescence intensity was measured using FACS caliber (BD Biosciences, USA)). A total of 10,000 events were acquired in each treatment group.

### Mitochondrial membrane depolarization assay

The integrity of the mitochondrial membrane was estimated by Rhodamine 123 (Rh 123) assay as described earlier [40]. Rh 123 is a cationic green fluorescent dye that can enter the mitochondrial matrix and the variation in the accumulation of Rh 123 in the cells is directly related to the change in the mitochondrial electrochemical potential (Δψ_M_). A decrease in the fluorescence of Rh 123 indicates a loss in the mitochondrial transmembrane potential and thus is a good method to identify mitochondrial damage. 6 × 10^5^ cells were seeded for the Rh 123 assay. After the completion of treatment, control and treated N9 cells were incubated with Rh 123 (0.3 μg/ml) for 20 min at 37 °C, followed by washing and resuspension in FACS buffer. A total of 10,000 events were acquired in each treatment group on a flow cytometer (BD FACS Calibur, BD Biosciences, USA) and the relative mean fluorescence intensity of Rh 123 was assessed. Staurosporine (1 μM)-treated N9 cells were used as positive control (data not shown).

### Caspase-1 activity assay

Levels of active caspase-1 were analyzed using caspase-1 activity assay kit (Millipore, USA, #21870) as per the manufacturer’s protocol. Briefly, around 3 × 10^6^ cells were pelleted and resuspended for 10 min in 50 μl chilled lysis buffer followed by centrifugation at 10,000*g* at 4 °C for 1 min. The supernatant containing cell lysate was quantified using BCA method. Two hundred micrograms of the cell lysates were incubated with 50 μl of 2× reaction buffer and the substrate (YVAD-p-Nitroaniline, at a final concentration of 200 μM) at 37 °C for 2 h followed by measurement of absorbance at 405 nm to quantify caspase-1 activity levels in different treatment groups. This assay is based upon the spectrophotometric detection of the chromophore p-nitroaniline (p-NA) after cleavage from the YVAD-pNA substrate because of the activation of caspase-1.

### Enzyme-linked immunosorbent assay (ELISA)

To quantify the levels of secreted IL-1β from different groups of N9 cells, ELISA was performed using mouse IL-1β ELISA kit (Biolegend, #432604) as per the manufacturer’s recommendations. Briefly, a rat monoclonal anti-mouse IL-1β capture antibody was coated in the 96-well plate overnight, followed by blocking for 1 h at room temperature (RT) and washing. For in vitro experiments, 1.5 × 10^6^ cells were seeded in 90 mm × 20 mm culture plates and media was collected after the completion of treatments. For in vivo experiments, BALB/c brain lysates were used. Control and treated samples (100 μl media supernatant for in vitro and 100 μg protein from the mice brain lysates for in vivo experiments) were incubated in these wells overnight at 4 °C. The samples were then incubated with biotin conjugated detection antibody for 1 h at RT, followed by addition of avidin-HRP substrate for 30 min. The absorbance was measured at 450 nm on a spectrophotometer (Biorad, Australia), and the concentrations were calculated using the IL-1β standard reference curves.

### Statistical analysis

Data are represented as the mean ± standard deviation (SD) from at least three independent experiments performed in triplicates (*n* = 3). The data was analyzed statistically by Student’s *t* test or one-way analysis of variance (ANOVA) followed by Holm-Sidak post hoc test. *P* value < 0.05 was considered significant. For in vivo treatments, a minimum of three mice were used in each group and experiments were repeated at least three times.

## Results

### Expression of IL-1β and HSP60 increase in various brain disorders

**Fig. 1:**
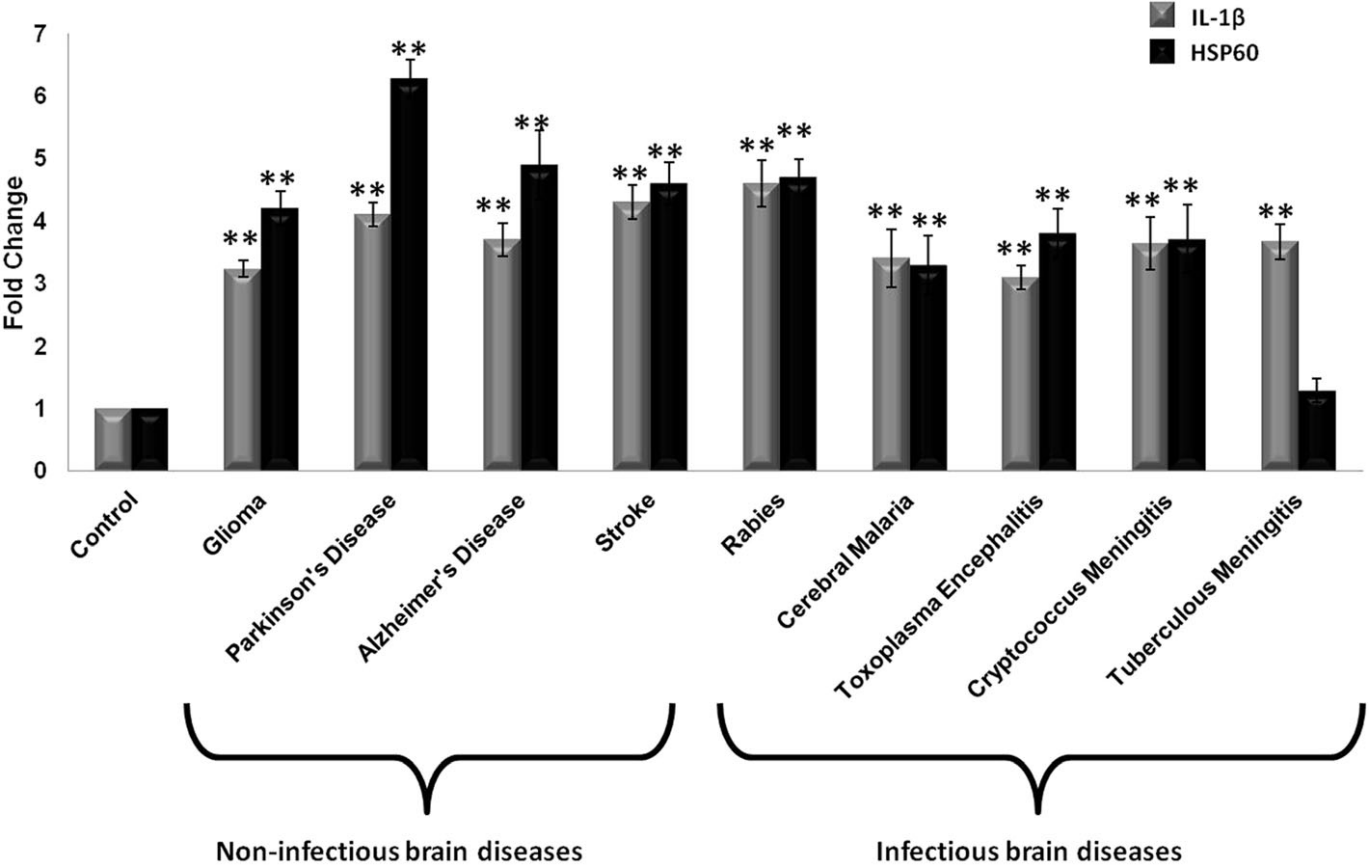
Expression of IL-1β and HSP60 increase in various human brain diseases. The levels of IL-1β and HSP60 gene expression were checked by qRT-PCR in frontal cortex of different neurological conditions and were compared with age-matched controls. For glioma, qRT-PCR was done with tissue sample and the expression of IL-1β and HSP60 were compared with that of control tissue. The transcript levels of the genes were normalized with the levels of GAPDH. The graph depicts pooled analysis of fold change in the levels of IL-1β and HSP60 in different brain diseases as compared with control brain. Data represented as mean ± SD from two different sets of experiments. The graph represents the pooled analysis of qRT-PCR data. **p < 0.01 in comparison to control condition

As IL-1β is considered to be the master regulator of the inflammation, its levels have been reported to be increased in various neurodegenerative disorders and brain infections as a result of microglial activation [9]. To confirm this, we compared the mRNA levels of IL-1β from sections of various human brain disorders including Alzheimer’s disease, Parkinson’s disease, stroke, rabies, tuberculous meningitis, cerebral malaria, toxoplasma encephalitis, and cryptococcus meningitis with control brain sections. For this, we performed qRT-PCR analysis from the FFPE human brain sections from the abovementioned neurological diseases and we found more than threefold increase in the levels of IL-1β in comparison to control sections (Fig. 1). In our previous study, we discovered that HSP60 plays a very important role in microglial inflammation by regulating underlying mechanism of IL-1β action. Therefore, we next determined the transcript levels of HSP60 in these diseased brain sections and found significant increase in HSP60 levels in almost all of these diseases when compared to control brain sections (Fig. 1). Similarly, levels of IL-1β and HSP60 get significantly elevated in glioma tissue in comparison to control (Fig. 1). Graph in Fig. 1 represents pooled data of all the qRT-PCR runs. This result signifies critical involvement of HSP60 in the pathogenesis of these neuronal disorders and neuronal infections besides IL-1β, and it might play an important role as an immunomodulatory molecule during neuronal infection and neurodegeneration.

### HSP60 is indispensable for IL-1β-mediated NF-κB phosphorylation

IL-1β after binding with its cognate receptor IL-1R1 can induce its own production by stimulating NLRP3 inflammasome complex [7]. It can also induce the phosphorylation of NF-κB and its nuclear localization in various cell types, which can signal the formation of inflammasome complex [41, 42]. Phosphorylation of NF-κB acts as a probing signal for the activation of NLRP3 inflammasome pathway which is responsible for endogenous IL-1β production by activated microglia. However, whether HSP60 plays a role in this endogenous IL-1β production via inflammasome pathway in microglial cells is not known. Hence, we set out to determine the effects of HSP60 on inflammasome pathway activation.

**Fig. 2:**
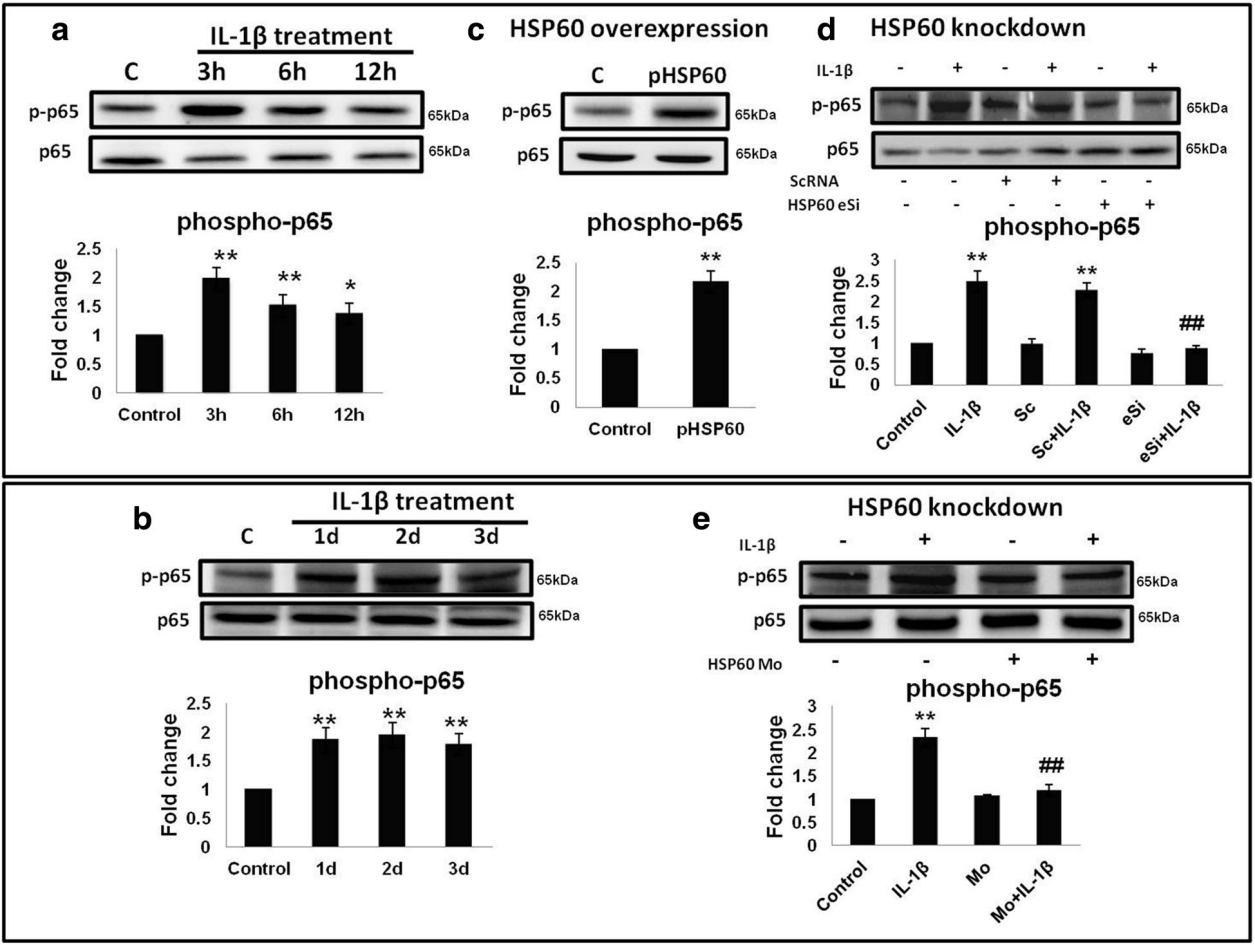
HSP60 is indispensable for IL-1β-mediated NF-κB phosphorylation. **a**, **b** Effect of IL-1β was checked on phosphorylation of p65-NF-κB in the cytoplasmic extracts of N9 cells (**a**) and mice brain (**b**). **c**, **d** Role of HSP60 in the induction of phosphorylation of p65 was checked in N9 cells by overexpression of HSP60 (**c**) and knockdown of HSP60 (**d**). **e** Effect of HSP60 knockdown with vivo-morpholino was checked in mice brain after IL-1β treatment for 3 days. Representative blots of three independent experiments are shown here. Bar diagrams below the blots represent quantification of the relative fold changes in phosphorylation of p65-NF-κB in comparison to control. The levels of p-p65-NF-κB were normalized with total p65-NF-κB. *p < 0.05, **p < 0.01 in comparison to control values. ##*p* < 0.01 in comparison to IL-1β treatment. Data represented as means ±SD of three independent experiments

For this, we first assessed the effect of IL-1β on NF-κB phosphorylation both in vitro and in vivo in the cytosolic extract. We found that IL-1β was able to significantly induce phosphorylation of p65-NF-κB both in vitro and in vivo (Fig. 2a, b). Next, we overexpressed HSP60 in N9 microglial cells and found that HSP60 overexpression was also able to induce p65-NF-κB phosphorylation in vitro (Fig. 2c). We then knocked-down HSP60 in N9 cells and treated cells with IL-1β for 3 h. To our surprise, IL-1β was not able to induce NF-κB phosphorylation after HSP60 reduction (Fig  2d). For in vivo knockdown of HSP60, mice were intracranially injected with HSP60-Mo. After the confirmation of specific knockdown of HSP60 by HSP60-Mo, the animals were divided into four groups and were treated with HSP60-Mo and IL-1β as described in the “Methods” section. Supporting our in vitro results, after reduction of HSP60 by HSP60-Mo, IL-1β was not able to induce phosphorylation of p65-NF-κB in vivo (Fig. 2e) also. This result confirms the crucial involvement of HSP60 in IL-1β-induced NF-κB phosphorylation.

### HSP60 plays a critical role in IL-1β-induced nuclear localization of NF-κB

**Fig. 3:**
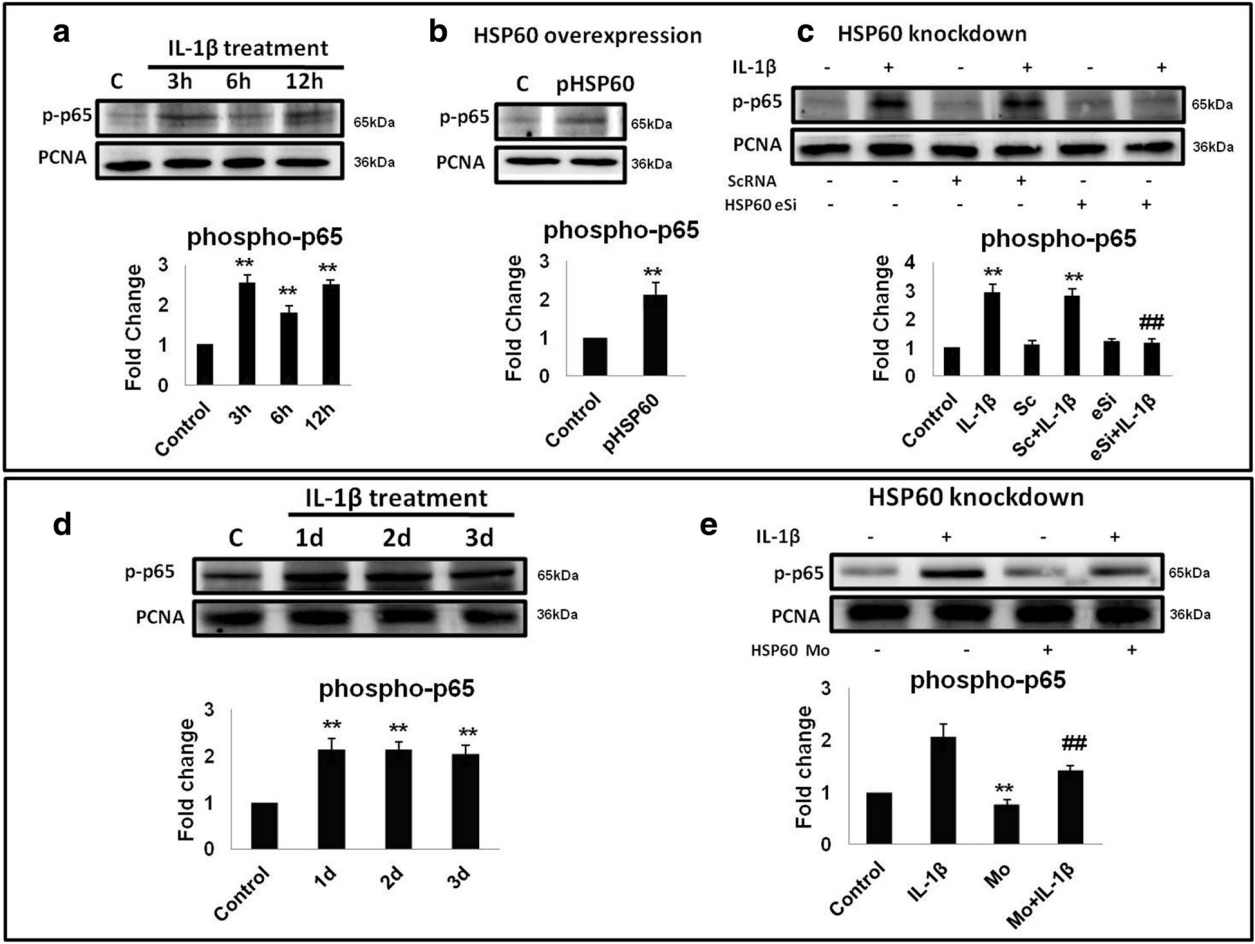
HSP60 plays a critical role in IL-1β-induced nuclear localization of pNF-κB. **a**, **d** Effect of IL-1β was checked on the nuclear localization of phospho-p65-NF-κB in the N9 cells (**a**) and mice brain (**d**). **b**, **c** Role of HSP60 in the induction of phosphorylation of p65-NF-κB was checked in N9 cells by overexpression of HSP60 (**b**) and knockdown of HSP60 (**c**). **e** Effect of HSP60 knockdown using vivo-morpholino was checked on nuclear localization of p65-NF-κB in mice brain after IL-1β treatment for different time periods. The levels of p65-NF-κB were normalized with the nuclear loading control PCNA protein levels. Representative blots of three independent experiments are shown here. Bar diagrams below the blots represent quantification of the relative fold changes in phosphorylated levels of NF-κB in comparison to control. *p < 0.05, **p < 0.01 in comparison to control values. ##*p* < 0.01 in comparison to IL-1β treatment. Data represented as means ±SD of three independent experiments

Phosphorylation of p65-NF-κB leads to its nuclear localization which is necessary for its function, i.e., regulation of expression of inflammatory genes. Hence, we checked the nuclear localization of phosphorylated p65-NF-κB (p-p65-NF-κB) upon IL-1β treatment in N9 microglial cells as well as BALB/c mice brains. We found that IL-1β treatment not only increases the phosphorylation of p65-NF-κB, but also leads to increase in the nuclear localization of phosphorylated p65-NF-κB, both in vitro and in vivo (Fig. 3a, d respectively). Simultaneously, we assessed the effect of HSP60 overexpression on the same and our results show that HSP60 overexpression in N9 microglial cells leads to increased nuclear localization of pNF-κB (Fig. 3b). To determine the role of HSP60 in IL-1β-induced nuclear localization of p-p65-NF-κB, we knocked-down HSP60 in N9 cells followed by IL-1β treatment and found that, after knockdown of HSP60, there was decrease in the nuclear localization of p-p65-NF-κB (Fig. 3c).We specifically knocked-down HSP60 in BALB/c mice brain using HSP60 vivo-morpholino and treated with IL-1β after 48 h of morpholino treatment. Our results show that in vivo knockdown of HSP60 led to decrease in the nuclear localization of NF-κB even after IL-1β treatment (Fig. 3e). These results suggest that HSP60 plays a critical role in the IL-1β-induced nuclear localization of pNF-κB.

### HSP60 regulates the expression of NLRP3 after IL-1β treatment

**Fig. 4:**
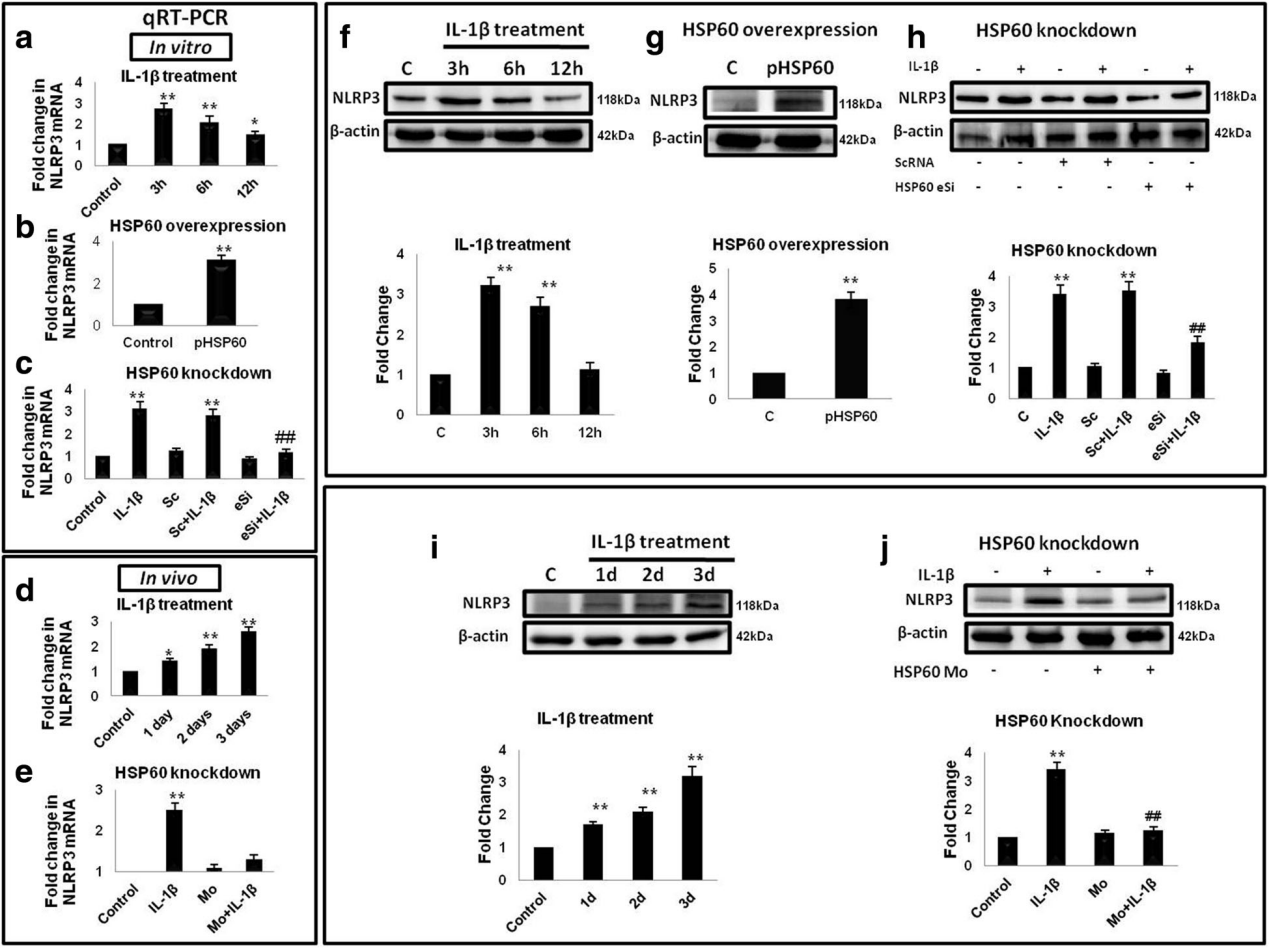
HSP60 regulates the expression of NLRP3 after IL-1β treatment. The left panel depicts the qRT-PCR analysis of NLRP3 gene (**a**–**e**) whereas the right panel shows the Western blot analysis (**f–j**). IL-1β treatment increased NLRP3 expression in vitro on both transcript level (**a**) and protein level (**f**). Similarly, NLRP3 expression was checked in vivo also through qRT-PCR (**d**) and Western blotting (**i**). HSP60 overexpression in microglial cells leads to increase in NLRP3 transcript level (**b**) and protein level (**g**). Effect of HSP60 knockdown on transcript levels (**c, e**) as well as protein levels (**h, j**) were observed in vitro and in vivo, respectively. Normalization of the transcript level was done with GAPDH while β-actin was used for the normalization of Western blots. For quantitative real-time PCR, each experiment was performed in triplicates. Representative blots of three independent experiments are shown here. Bar graphs below the blots represent the quantification of protein levels. *p < 0.05, **p < 0.01 in comparison to control values. ##*p* < 0.01 in comparison to IL-1β treatment. Data represented as mean ± SD of three independent experiments

Nuclear localization of pNF-κB facilitates the activation of NLRP3 inflammasome pathway by inducing the transcription of NLRP3 gene and pro-IL-1β [41, 43]. We also observed that IL-1β induces the phosphorylation and nuclear localization of NF-κB in a HSP60-dependent manner (Figs. 2 and 3); therefore, we next explored the role of HSP60 in IL-1β-induced NLRP3 expression through qRT-PCR and Western blot. For this, we first assessed the effect of IL-1β on NLRP3 expression and we found that IL-1β treatment significantly increases mRNA and protein levels of NLRP3 both in vitro (Fig. 4a, f) and in vivo (Fig. 4d, i). To investigate the role of HSP60, we overexpressed HSP60 gene in N9 cells as described in the “Methods” section. HSP60 induced the expression of NLRP3 both at transcript and protein levels (Fig. 4b, g), and its downregulation reduced the NLRP3 expression even after IL-1β treatment (Fig. 4c, h). Similarly, in BALB/c mice brain samples, IL-1β treatment increases expression of NLRP3 (Fig. 4d, i); however, the expression of NLRP3 did not increase after IL-1β treatment in the HSP60 vivo-morpholino-treated group and were comparable to control group (Fig. 4e, j). These results show that HSP60 expression is critical for IL-1β-induced NLRP3 expression.

### HSP60 induces mitochondrial damage and oxidative stress

**Fig. 5:**
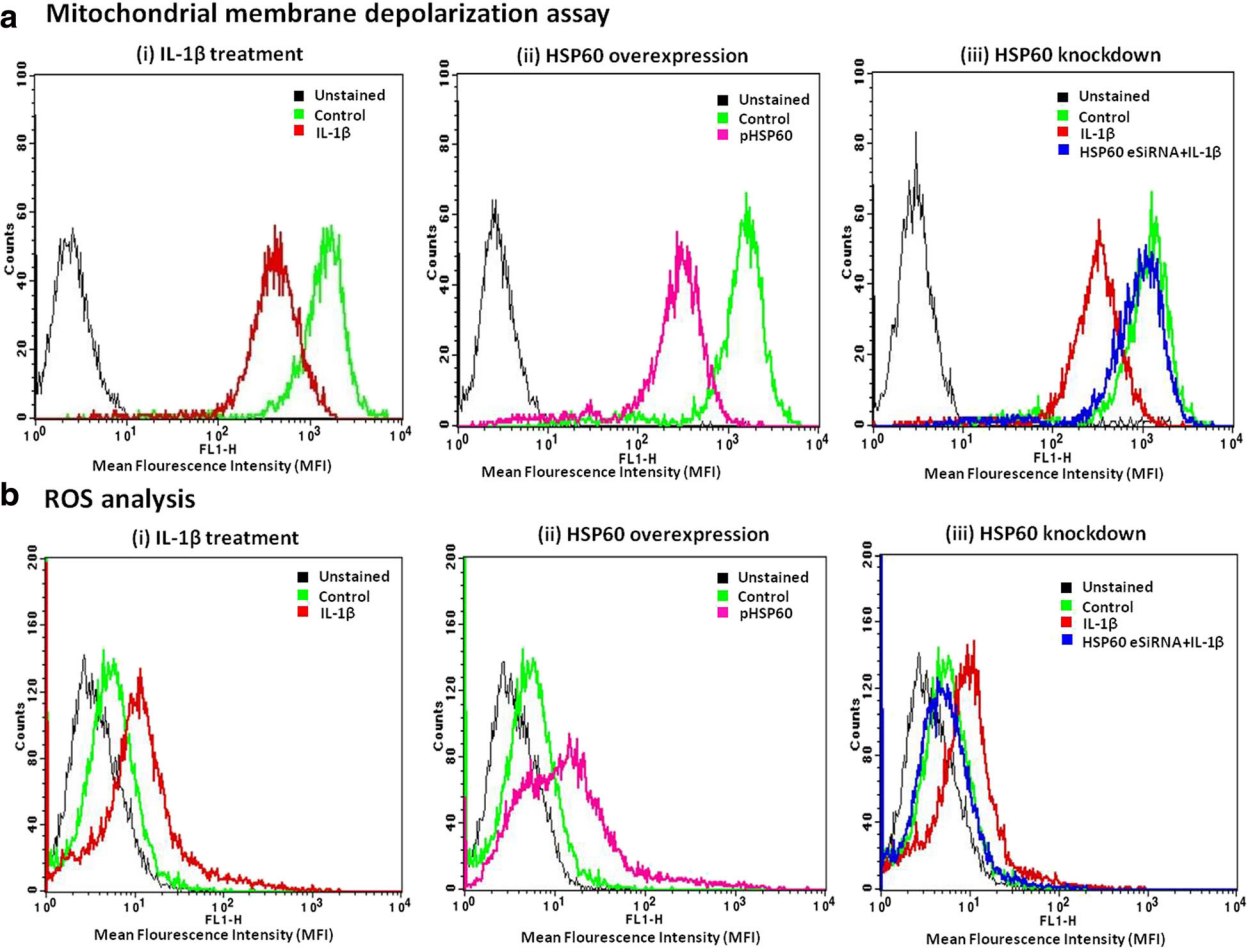
HSP60 induces mitochondrial damage and oxidative stress. **a** Mitochondrial damage was assessed in N9 cells using FACS by the quantification of mitochondrial membrane potential using Rhodamine 123 dye (upper panel). Histograms show the effect of IL-1β (i), effect of HSP60 overexpression (ii), and HSP60 knockdown (iii) on mitochondrial membrane potential. **b** ROS generation in N9 microglial cells was assessed by FACS using DCFDA (lower panel). Histograms in the lower panel show the effect of IL-1β (i), effect of HSP60 overexpression (ii), and HSP60 knockdown (iii) on ROS generation by microglia. Data show that HSP60 knockdown lead to significant reduction in mitochondrial depolarization and ROS generation by microglia (*p* < 0.01). For FACS analysis, each experiment was performed in triplicates. Results are representative of three independent experiments

NLRP3 inflammasome is activated in response to a variety of stimuli, supporting the fact that different signals induce similar downstream events that are sensed by NLRP3. The widely studied mechanisms of NLRP3 activation include the mitochondrial damage leading to the decrease in mitochondrial membrane potential and generation of mitochondrial reactive oxygen species (ROS) [44]. To assess the effect of IL-1β treatment and HSP60 modulation on mitochondrial membrane potential, we performed Rhodamine 123 (Rh 123) assay. We observed that IL-1β treatment (for 3 h) as well as HSP60 overexpression led to decrease in the mitochondrial membrane potential in microglial cells, indicating the mitochondrial damage (Fig. 5a (i-ii)). Cells with HSP60 knockdown do not display mitochondrial damage as mitochondrial membrane potential was comparable to control cells even after IL-1β treatment (Fig. 5a (iii)).

Literature suggests that IL-1β increases ROS generation in microglia [45]. We also confirmed increase in ROS generation in N9 cells after IL-1β treatment (Fig. 5b (i)). We found that ROS generation in N9 cells increased up to 3.5-fold after 3 h of IL-1β treatment in comparison to untreated control cells. Further, to determine the effects of HSP60 on ROS, we overexpressed and knocked-down HSP60 in N9 cells. Overexpression of HSP60 hugely induces ROS generation (6.2-fold in comparison to control) (Fig. 5b (ii)) whereas its knockdown drastically reduces the effect of IL-1β on ROS generation (Fig. 5b (iii)) and ROS levels become comparable to that of control cells.

### Role of HSP60 in IL-1β-induced caspase-1 activation

**Fig. 6:**
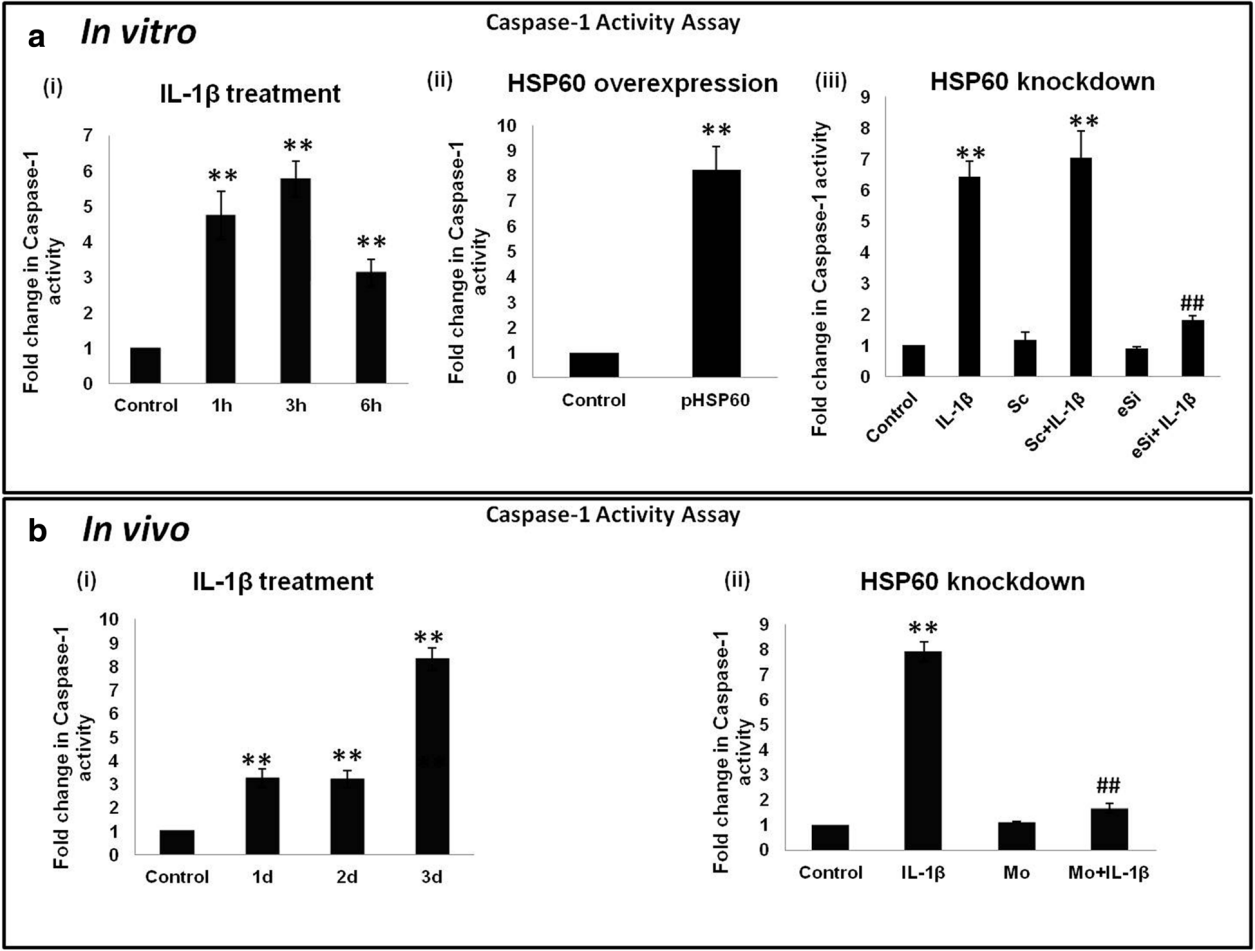
Role of HSP60 in IL-1β-induced caspase-1 activation. Caspase-1 activity in the N9 cells (upper panel) and mice brain (lower panel) was assessed by caspase-1 activity kit. **a** Bar graphs in the upper panel show the effect of IL-1β (i), effect of HSP60 overexpression (ii), and HSP60 knockdown (iii) on caspase-1 activity in N9 cells. **b** Bar graphs in the lower panel show the effect of IL-1β (i) and HSP60 knockdown (ii) on caspase-1 activity in mice brain. Each experiment was performed in triplicates. Data represented as mean ± SD of three independent experiments (*n* = 3). *p < 0.05; **p < 0.01 in comparison to control values and ##*p* < 0.01 in comparison to IL-1β treatment

NLRP3 inflammasome complex, when activated in response to different cell damage and/or, stress stimuli, leads to the cleavage of pro-caspase-1 to caspase-1 which is also known as interleukin-converting enzyme (ICE). Formation of caspase-1 from pro-caspase-1 is the executioner step of inflammasome pathway which is responsible for the maturation of IL-1β from pro-IL-1β. We analyzed the levels of active caspase-1, both in vitro and in vivo. Our in vitro data show that both IL-1β treatment and HSP60 overexpression increased the activity of caspase-1 in N9 cells by 5.8 folds and 8.1 folds respectively (Fig. 6a (i-ii)). However, HSP60 knockdown does not allow increase in caspase-1 activity even after IL-1β treatment (Fig. 6a (iii)). Further, our in vivo results recapitulate the in vitro results. In in vivo conditions, IL-1β increases the levels of active caspase-1 via HSP60 as the knockdown of HSP60 reduces IL-1β-induced active caspase-1 levels (Fig. 6b (i) and (ii)). This result suggests that HSP60 plays an important role in caspase-1 activation.

### HSP60 critically regulates microglial IL-1β production both in vitro and in vivo

**Fig. 7:**
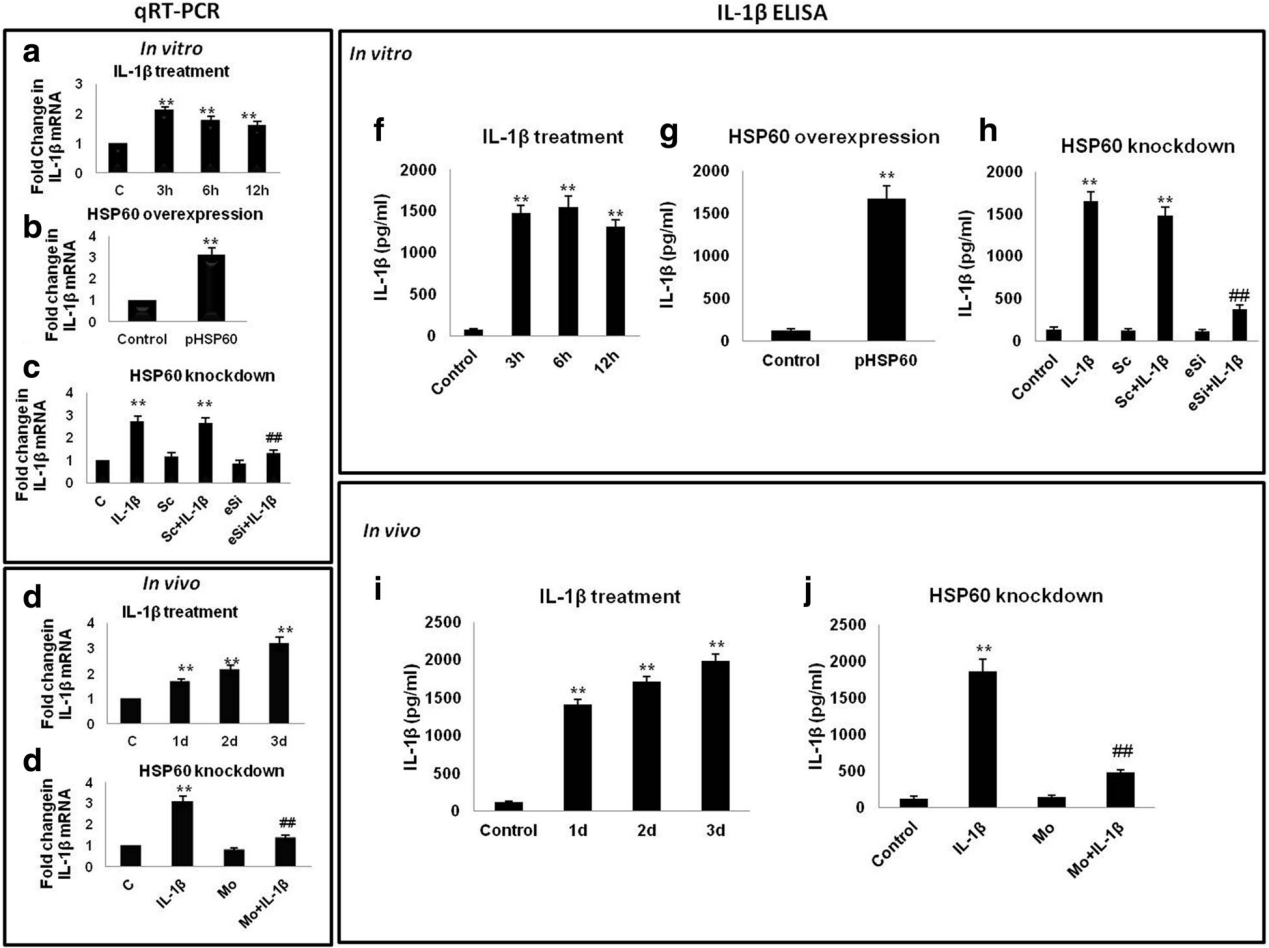
HSP60 critically regulates microglial IL-1β production both in vitro and in vivo*.* Expression of IL-1β gene and its secretion by activated microglia was checked by qRT-PCR and ELISA respectively. Left panel depicts the qRT-PCR analysis of IL-1β gene (**a–e**) while right panel shows the IL-1β ELISA (**f–j**). IL-1β treatment increases its own expression in vitro (**a**) and induces its own secretion also (**f**). Similarly, IL-1β expression was checked through qRT-PCR (**d**) and ELISA (**i**) in vivo. **b**, **g** HSP60 overexpression in microglia leads to increase in transcript level of IL-1β (**b**) and its secretion from microglia (**g**). Effect of HSP60 knockdown on transcript levels (**c, e**) as well as secreted levels of IL-1β (**h, j**) was also observed in vitro and in vivo, respectively. Normalization of the transcript level was done with GAPDH. Both qRT-PCR analysis and ELISA were performed in triplicates for each experiment. Data shown is representative of three independent experiments (*n* = 3). *p < 0.05, **p < 0.01 in comparison to control values. ##*p* < 0.01 in comparison to IL-1β treatment. Data represented as mean ± SD of three independent experiments

To determine whether endogenous IL-1β production is mediated by HSP60, we finally checked the effect of HSP60 on endogenous IL-1β production in response to IL-1β treatment both in vitro (N9 cells) and in vivo (BALB/c mice brains). We assessed the levels of expression of IL-1β through qRT-PCR and its secretion by ELISA. We observed that IL-1β treatment and HSP60 overexpression increases IL-1β production and it gets secreted by microglial cells in vitro (Fig. 7a, b, f, g respectively). Knocking down HSP60 in N9 cells compromised the expression and secretion of IL-1β even after IL-1β treatment (Fig. 7c, h). Similarly, in BALB/c mice brains also, IL-1β induces its own production in vivo (Fig. 7d, i,). However, IL-1β treatment in mice brain preceded by HSP60 downregulation was unable to induce IL-1β production (Fig. 7e, j). These results show that HSP60 indeed plays a critical role in IL-1β inducing its own production by activated microglia via regulating NLRP3 inflammasome pathway.

### Japanese encephalitis virus (JEV)-induced IL-1β production by activated microglia is regulated by HSP60

**Fig. 8:**
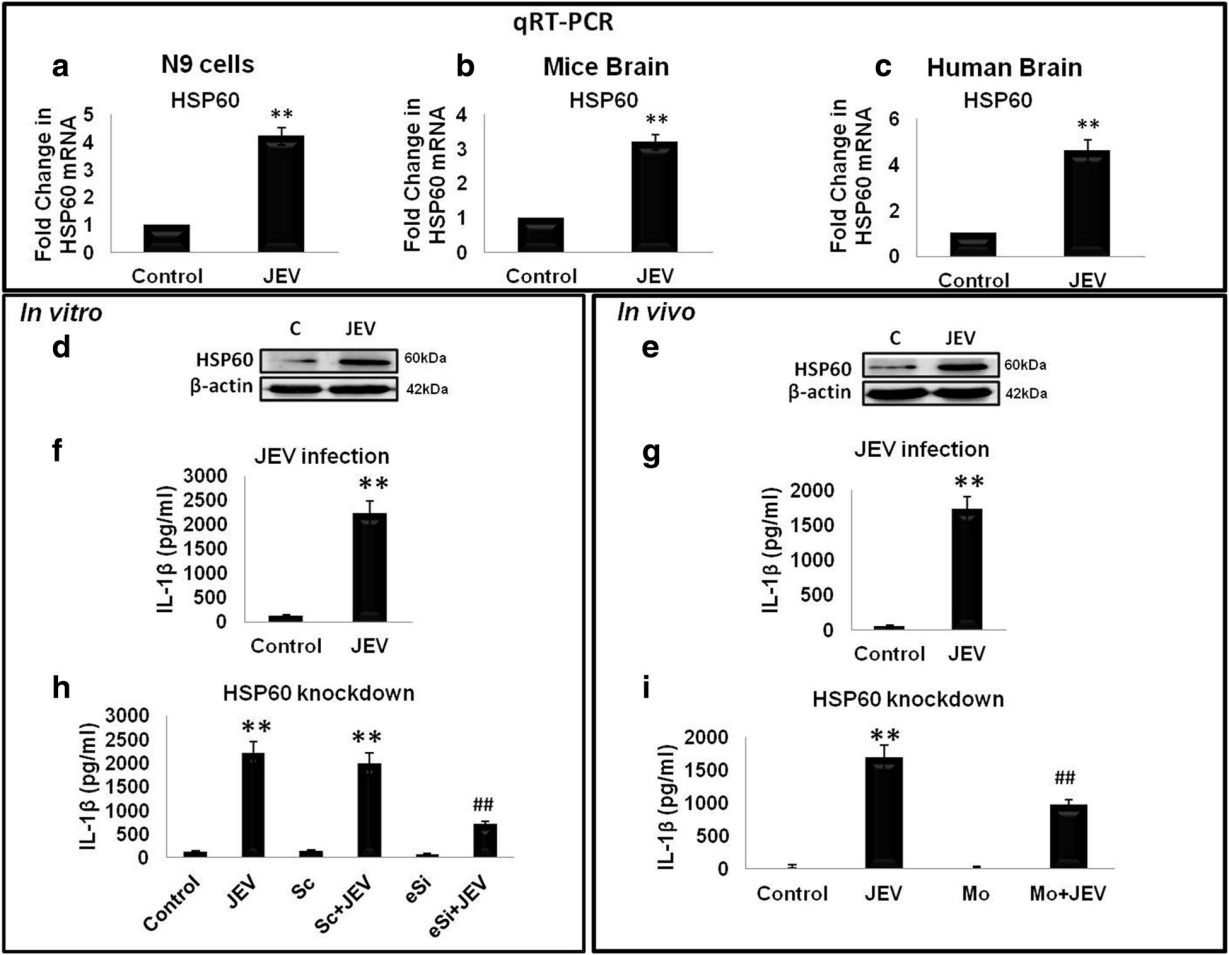
Japanese encephalitis virus (JEV)-induced IL-1β production by activated microglia is regulated by HSP60. Upper panel depicts the qRT-PCR data. **a–c** JEV infection increases HSP60 both at RNA level (**a**, **b**) and protein level (**d**, **e**) in N9 cells and mice brains respectively. Protein levels of HSP60 in the Western blot were normalized with β-actin levels while transcript expression of HSP60 was normalized with GAPDH expression. **c** Effect of JEV infection on the transcript level of HSP60 was also assessed in FFPE human brain sections infected with JEV and were compared with the control brains. **f**, **g** JEV infection increases IL-1β secretion both in vitro (**f**) and in vivo (**g**) which were analyzed using ELISA. **h**, **i** HSP60 knockdown leads to decrease in the IL-1β secretion as assessed by ELISA in N9 cells (**h**) and mice brain lysate (**i**). Both qRT-PCR and ELISA were performed in triplicates for each experiment. Data represented as mean ± SD of three independent experiments (*n* = 3). *p < 0.05, **p < 0.01 in comparison to control values and ##*p* < 0.01 with respect to JEV-infected values

JEV is a common cause of acute and epidemic viral encephalitis. JEV infection is associated with microglial activation resulting in the production of pro-inflammatory cytokines. As our data in the previous section show that HSP60 regulates IL-1β production (Fig. 7), hence, we were curious to explore whether it regulates IL-1β production during JEV infection also, which is a very good model to study neuroinflammation. We first determined levels of HSP60 in JEV-infected N9 cells, mice brains, and FFPE human brain sections through qRT-PCR and found that JEV infection was able to significantly increase the expression of HSP60 transcripts (Fig. 8a–c). Protein levels of HSP60 also significantly increased in JEV-infected N9 cells and mice brain as compared to control (Fig. 8d, e). Literature suggests that JEV infection induces IL-1β production by stimulating the NLRP3 inflammasome pathway [29, 30]. We tested this notion and confirmed induction of IL-1β in vitro and in vivo upon JEV infection through ELISA (Fig. 8f, g). Next, to explore the role of HSP60 in JEV-induced IL-1β production, we knocked-down HSP60 both in vitro (N9 cells) and in vivo (BALB/c mice brain) as described in the “Methods” section. To our surprise, knockdown of HSP60 was sufficient to reduce JEV infection-mediated IL-1β production (Fig. 8h, i). These results suggest that downregulation of HSP60 leads to alteration of inflammasome pathway which hampers JEV-induced IL-1β production by activated microglia.

### Downregulation of HSP60 results in reduction in JEV-induced microglial inflammation

**Fig. 9:**
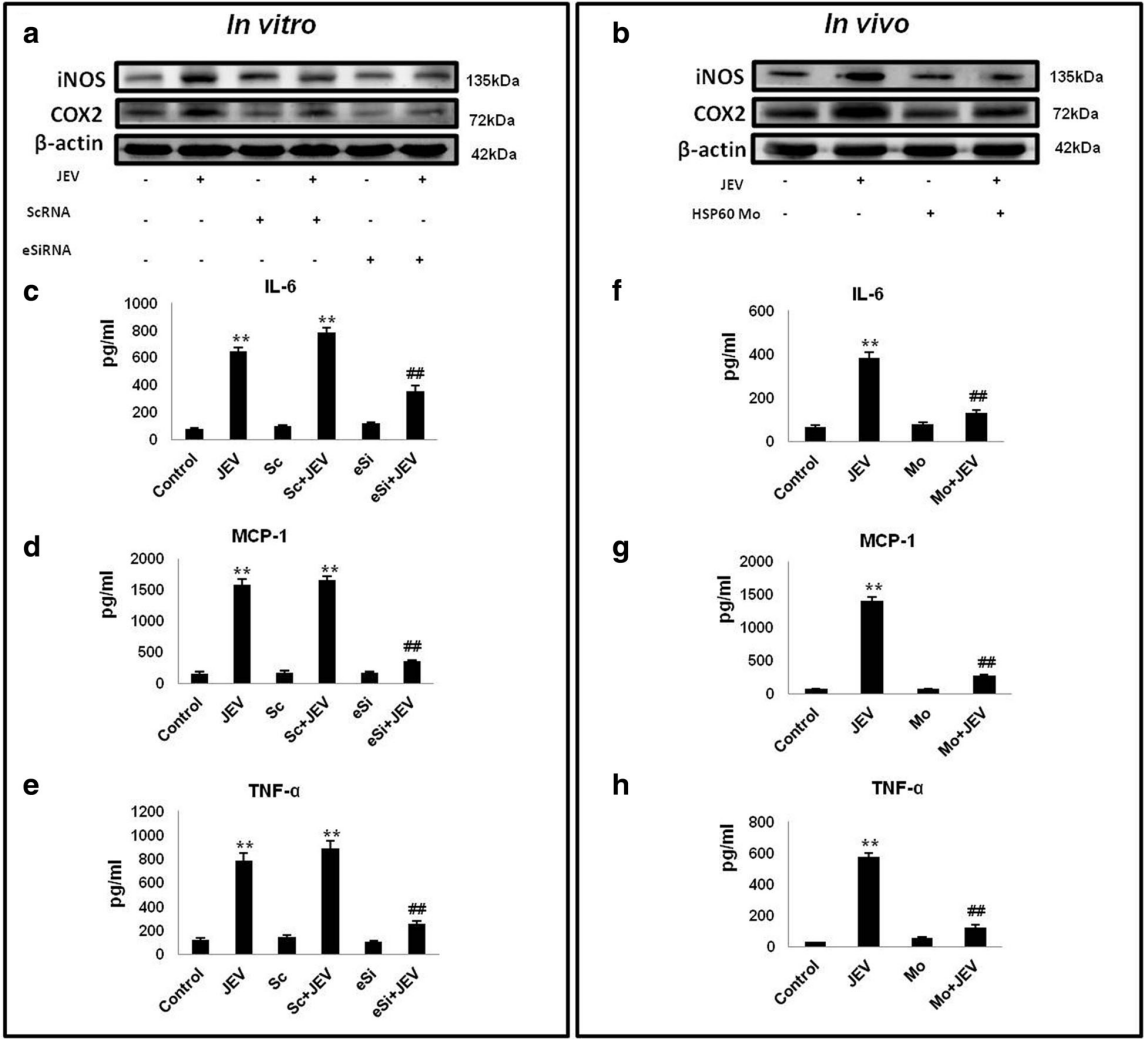
Downregulation of HSP60 reduces JEV-induced microglial inflammation. The left panel shows the effect of HSP60 knockdown with specific eSiRNA on JEV-induced microglial inflammation in N9 cells, while the right panel shows the effect of HSP60 knockdown using HSP60 vivo-morpholino in JEV-infected mice brains. **a**, **b** Western blots of iNOS and COX2 after HSP60 knockdown during JEV infection in N9 cells and mice brain respectively. Protein levels of iNOS and COX2 were normalized with the β-actin levels. The blots are representative of three independent experiments. **c–h** CBA of pro-inflammatory markers was performed to assess the role of HSP60 in JEV-induced microglial inflammation. Bar graphs show quantification of the cytokines levels in N9 cells (**c–e**) and in mice brains (**f–h**). Cytokine bead array was performed in triplicates for each experiment. For animal experiments, at least three mice were used in each group. Data represented as mean ± SD of three independent experiments (*n* = 3). *p < 0.05, **p < 0.01 in comparison to control values. ##*p* < 0.01 with respect to JEV-infected values

HSP60 knockdown results in a decrease in IL-1β production after JEV infection both in vitro and in vivo (Fig. 8h, i) and as IL-1β is the main cytokine involved in microglial activation, we speculated that reducing HSP60 levels may also ameliorate JEV-induced inflammation. To test this, we assessed the levels of important pro-inflammatory enzymes (iNOS and COX2) by Western blotting (Fig. 9a, b) and performed cytometric bead array (CBA) for measuring the levels of pro-inflammatory cytokines (TNF-α, MCP-1, and IL-6) in N9 cells as well as BALB/c mice brains after JEV infection (Fig. 9c–h). We observed that downregulation of HSP60 both in vitro and in vivo leads to reduction of these pro-inflammatory markers after JEV infection.

### Knockdown of HSP60 leads to increased survival and amelioration of behavioral deficits in JEV-infected mice

**Fig. 10:**
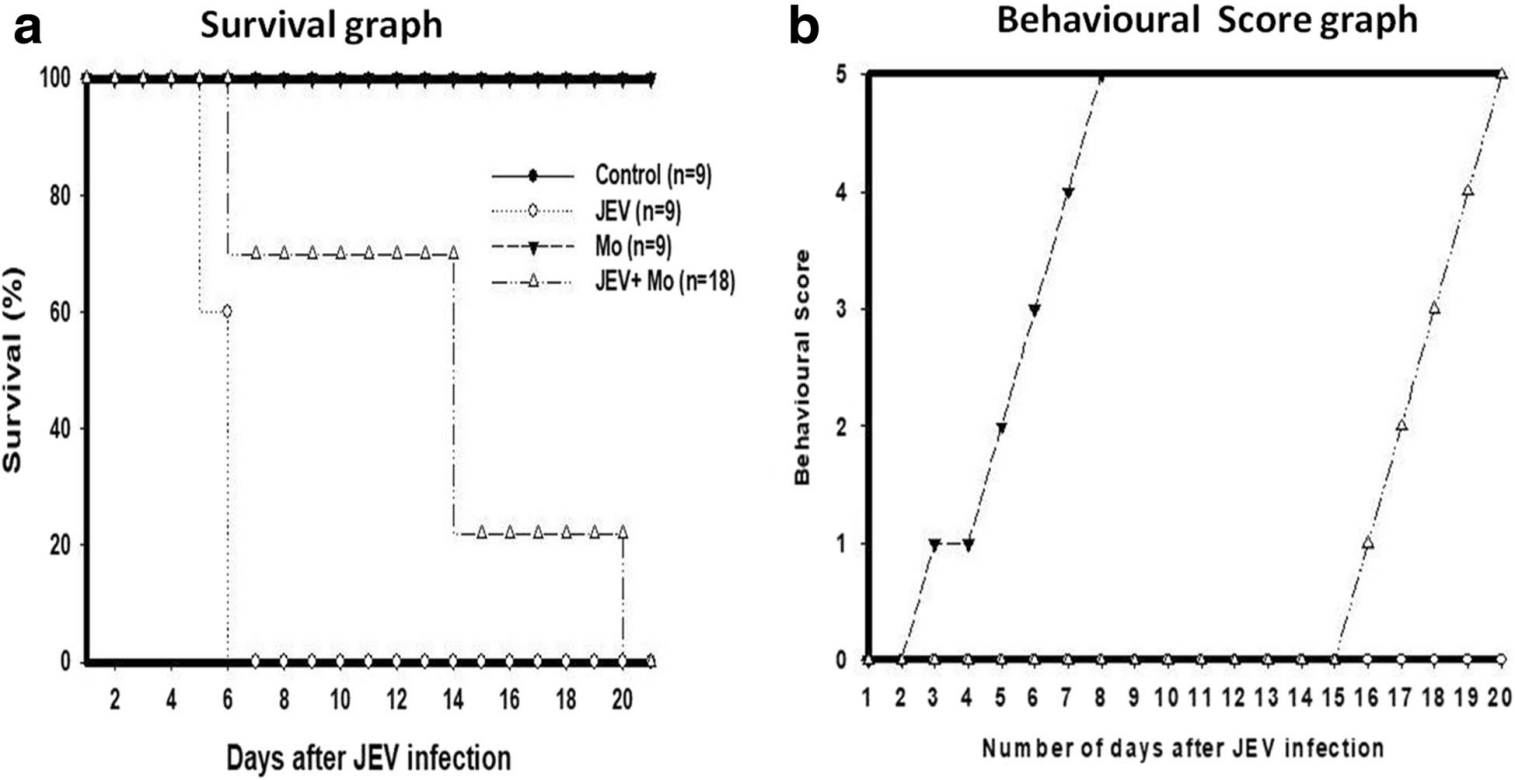
Effect of HSP60 knockdown on the survival and behavior of the JEV-infected mice. **a** Survival plot showing increase in the survival of the mice after reduction in the inflammation by knockdown of HSP60. **b** Behavioral score plot shows delayed onset of the symptoms of JEV infection. Different scores were given for the behavior of the mice based on the symptoms. 0 = No pilorection; No body stiffening; No restriction of movement; No paralysis; No body tremor. 1 = Pilorection; No body stiffening; No restriction of movement; No paralysis; No body tremor. 2 = Pilorection; body stiffening; No restriction of movement; No paralysis; No body tremor. 3 = Pilorection; body stiffening; restriction of movement; No paralysis; No body tremor. 4 = Pilorection; body stiffening; restriction of movement; paralysis; No body tremor. 5 = Pilorection; body stiffening; restriction of movement; paralysis; body tremor. Data shown is representative of three different independent experiments and ‘*n*’ represents the number of animals in each group

As knocking down HSP60 reduced the inflammation in JEV-infected mice, so we questioned what would be the effect of HSP60 on survival of JEV-infected mice. We observed that in BALB/c mice knockdown of HSP60 not only reduced the level of inflammatory markers, but was able to significantly increase the survival of the infected animal also. Animals pretreated with HSP60 vivo-morpholino before JEV infection showed delayed onset of symptoms and the survival was significantly increased than those of JEV-infected group (more than 10 days after the death of JEV-infected mice) (Fig. 10a). In addition, the mice from JEV-infected groups showed behavioral deficits after the onset of symptoms (viz. tremor, hind limb paralysis, motor deficit) which were improved and delayed after knockdown of HSP60 (Fig. 10b). We compared the behavior of the HSP60-Mo + JEV-infected mice with only JEV-infected mice by giving scores based on the visible symptoms as shown in the graph. These results suggest HSP60 reduces the inflammation during JEV infection that leads to delayed infection and increased survival of the organism. Thus, our results illuminate HSP60 as a novel therapeutic target against JEV infection.

## Discussion

In our previous study, we identified and demonstrated that HSP60 critically regulates IL-1β-induced microglial inflammation through TLR4-p38MAPK axis [10]. Despite the plethora of literature on the master regulator of inflammation viz. IL-1β, a comprehensive mechanism underlying its constitutive production in activated microglia remains elusive. Therefore, to explore the underlying mechanism, we investigated the effect of HSP60 on NLRP3 inflammasome pathway that induces IL-1β production through caspase-1 activation. In this study, for the first time, we present in vitro and in vivo evidence to demonstrate that HSP60 functions as a potent inducer of NLRP3 inflammasome activation and IL-1β production in N9 microglial cells and mice brain tissues. Furthermore, we demonstrate that HSP60 induces mitochondrial stress and ROS generation and activates caspase-1 to enhance sustained IL-1β production.

Our data show that IL-1β expression increases in various non-infectious as well as infectious brain inflammatory diseases. This result is in concordance with various previous studies which show that IL-1β gets upregulated in response to neurodegeneration and CNS infection [46–51]. This suggests that IL-1β is a critical inflammatory factor involved in neuroinflammatory and neurodegenerative diseases. In addition, we found increased levels of HSP60, equivalent to IL-1β, in almost all the diseased human brain tissues we investigated. Various studies indicate that HSP60 levels increase in neuroinflammatory and neurodegenerative diseases [52–54]. These studies along with our results signify that HSP60, besides acting as mitochondrial chaperone and a stress molecule also function as an immunomodulator. A few studies have also shown the involvement of heat shock proteins and other stress-induced proteins in cytokine production [55, 56].

Our results along with previous studies suggest that IL-1β, after being secreted by activated microglia, induces its own production by stimulating NLRP3 inflammasome complex in glioma cells, monocytes, and other cell types [7, 49]. It has been established that IL-1β can induce death through mitochondrial dysfunction in chondrocyte cells [57, 58]. Mitochondrial damage can also trigger the activation of NLRP3 inflammasome, which propagates endogenous IL-1β production by microglia [59]. Here, we also corroborate these findings by demonstrating that IL-1β treatment in microglial cells leads to increased phosphorylation and nuclear localization of NF-κB, which in turn upregulates the transcription of pro-IL-1β and NLRP3 genes. In addition, IL-1β treatment induces mitochondrial damage and thus leads to ROS generation in microglia. All these driving factors lead to the activation of NLRP3 inflammasome complex. However, the role of HSP60 in this pathway was not elucidated out.

HSP60 acts as an immunomodulatory molecule as it can activate antigen-presenting cells of the immune system as an auto-immunogen at the site of inflammation [60, 61]. Further, it gets upregulated in response to mitochondrial impairment and is considered to be an indicator of mitochondrial stress. Evidence indicate HSP60 as a connecting link between mitochondrial stress and inflammation in diabetes mellitus [25]. This led to the framework of our study and prompted us to explore the role of HSP60 in endogenous IL-1β production by activated microglia. Here, we show the regulatory role of HSP60 in mitochondrial and NLRP3 inflammasome pathway. HSP60 plays an important role in NLRP3 inflammasome activation as our data show that knocking down HSP60 leads to decreased phosphorylation of NF-κB, scanty ROS production, reduced NLRP3 levels, and finally abrogated inflammation.

We further established that IL-1β-induced NLRP3 inflammasome activation is ameliorated after reduction of HSP60. Increase in the activity of caspase-1 is the executioner step in the NLRP3 inflammasome pathway. Our results show that knockdown of HSP60 both in vitro and in vivo led to decrease in caspase-1 activity, which is also reflected by reduced IL-1β production. Hence, HSP60 on the one hand induces mitochondrial stress leading to reduction in mitochondrial membrane potential and elevates ROS generation, and on the other hand, it increases the phosphorylation and nuclear localization of NF-κB leading to upregulation of NLRP3, pro-IL-1β, and other inflammatory genes, thus linking mitochondrial stress to inflammation. These results further delineate the inflammatory pathway induced by IL-1β via HSP60 by stimulating TLR4-p38 MAPK axis [10]. Besides these results, some questions still remain to be answered, for example, how HSP60 induced NF-κB phosphorylation, does it interact with IκB (regulatory element of NF-κB), or is it a p38-dependent or p38-independent pathway. Very recently, p38 has been shown to activate inflammasome in human keratinocytes [62]. However, neuroinflammation is a complex biochemical process and therefore further investigation is warranted to have a conclusive answer.

JEV, a neurotropic virus belonging to *Flaviviridae* family, invades the CNS after the initial infection of peripheral tissues [63]. JEV infection is a common cause of acute and epidemic viral encephalitis, causes the robust microglial activation, and increases the IL-1β production that increases the severity of infection [28–30]. We observed significant increase in HSP60 expression during JEV infection (Fig. 8). However, contrary to our finding, decreased expression of HSP60 after JEV infection has been shown in 4–6-week-old mice pups, and this contradiction might be due to the age difference of mice [64]. Our results further show that specific knockdown of HSP60 during JEV infection led to reduction in IL-1β levels and inflammation in N9 microglial cells as well mice brains. In addition, we also observed increased survival and delayed onset of the symptoms of JEV infection after knockdown of HSP60. The plausible reason for this delayed onset of symptom and increased survival might be the reduction in inflammation due to knockdown of HSP60. Decreasing inflammation in case of virus infection by anti-inflammatory drugs leads to increased survival of the organism which has already been reported [65, 66]. In case of JEV, treatment with minocycline, an anti-inflammatory drug, results in increased survival [67]. A recent study indicates that transient degradation of mitochondrial HSP60 during early hours of rotavirus-SA11 infection results in delayed apoptosis [68]. HSP60 has already been proposed to be a potential drug target against human hepatitis B virus (HBV) as downregulation of HSP60 in infected cells blocks replication of HBV [22].

**Fig. 11:**
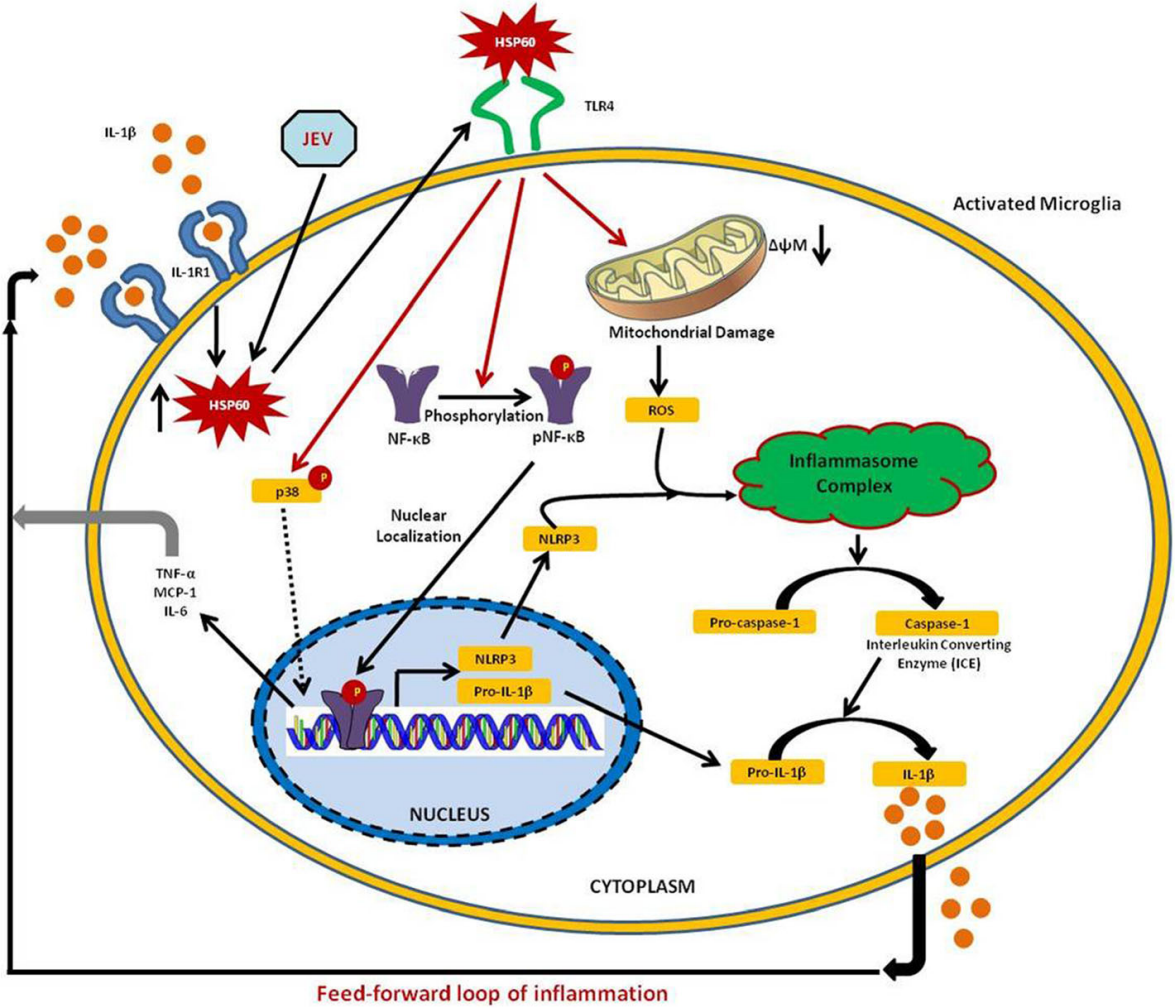
Schema of signaling pathway involved in HSP60-mediated NLRP3 inflammasome activation and subsequent IL-1β production. IL-1β induces its own production by the activated microglia in a HSP60-dependent manner. HSP60, after being upregulated by IL-1β, gets secreted outside and binds with TLR4 of the microglia to activate p38 MAPK [10]. Binding of HSP60 with TLR4 facilitates NF-κB phosphorylation, mitochondrial damage, and ROS generation and finally activates NLRP3 inflammasome leading to IL-1β production. JEV also augments HSP60 production and thus influences inflammasome complex to induce a consecutive expression of IL-1β and, in turn, induces an exaggerated immune response

To summarize, our current study establishes that HSP60, a mitochondrial chaperone and immunomodulatory molecule, regulates endogenous IL-1β production by inducing mitochondrial stress and activating NLRP3 inflammasome pathway in microglia. For the first time, we establish that downregulating HSP60 decreases IL-1β production and inflammation in JEV infection. Thus, we hereby propose a feed-forward loop of inflammation where HSP60 is increased in microglia in response to harmful stimuli and in turn stimulates inflammasome complex which results in consecutive microglial activation (Fig. 11). This study thus provides the understanding of a complex signaling mechanism involved in neuroinflammation and also suggests HSP60 as a potential therapeutic target for the amelioration of various neuroinflammatory and neurodegenerative diseases.

## Conclusions

The findings in the present study strongly suggest the important role of HSP60 as an immunomodulatory molecule in neuroinflammation. Our results show that HSP60 levels increase in microglia upon sensing stress and danger stimuli viz. IL-1β treatment and JEV infection, respectively. After being upregulated, HSP60 exacerbates neuroinflammation by stimulating IL-1β production by the activated microglia by inducing NLRP3 pathway. On the one hand, it induces phosphorylation and nuclear localization of NF-κB, leading to upregulation of NLRP3 and IL-1β expression, and on other hand, it induces mitochondrial damage and ROS generation to trigger the activation of NLRP3 inflammasome complex. Knocking down HSP60 leads to decrease in the IL-1β secretion by microglia, and as IL-1β is the key mediator of inflammation in CNS, its reduction leads to the amelioration of inflammation. Our results also manifest that reduction of HSP60 leads to decreased inflammation and increased survival in the JEV-infected mice. We here provide the first evidence of the regulatory involvement of HSP60 in IL-1β production by the activated microglia and its role in JEV infection.

## Additional file

Additional file 1: **Table S1.** Primers list used for quantitative real-time PCR analysis of different genes. **Figure S1.** Confirmation of HSP60 knockdown by vivo morpholino in mice brain. **Figure S2.** The overexpression of HSP60 was confirmed by Western blot. **Figure S3.** JEV induces inflammation in N9 murine microglial cells at MOI 2. (DOCX 1066 kb)
