# Correction to: HSP60 critically regulates endogenous IL-1β production in activated microglia by stimulating NLRP3 inflammasome pathway

**DOI:** 10.1186/s12974-018-1355-6

**Published:** 2018-11-15

**Authors:** Shalini Swaroop, Anita Mahadevan, Susarla Krishna Shankar, Yogita K. Adlakha, Anirban Basu

**Affiliations:** 1 National Brain Research Centre, Manesar, Haryana, 122052 India; 2 Department of Neuropathology, National Institute of Mental Health and Neurosciences, Bangalore, India

## Correction

Upon publication of the original article [1], it was noticed that there is an error in Fig. 10, the dialog box in panel (b) was missing. The correct Fig. 10 is shown below.

**Fig. 10:**
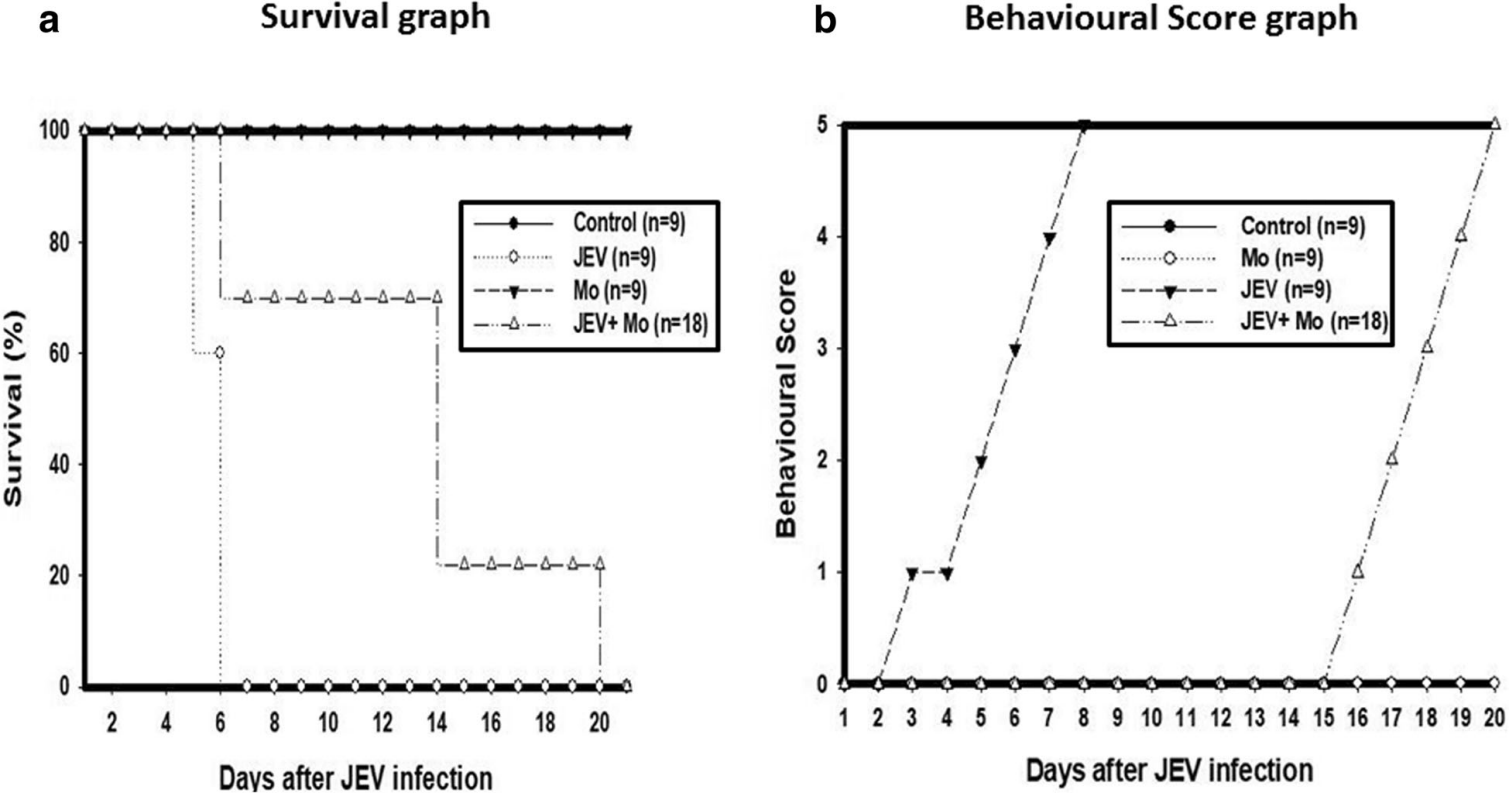
Effect of HSP60 knockdown on the survival and behavior of the JEV-infected mice. **a** Survival plot showing increase in the survival of the mice after reduction in the inflammation by knockdown of HSP60. **b** Behavioral score plot shows delayed onset of the symptoms of JEV infection. Different scores were given for the behavior of the mice based on the symptoms. 0 = No pilorection; No body stiffening; No restriction of movement; No paralysis; No body tremor. 1 = Pilorection; No body stiffening; No restriction of movement; No paralysis; No body tremor. 2 = Pilorection; body stiffening; No restriction of movement; No paralysis; No body tremor. 3 = Pilorection; body stiffening; restriction of movement; No paralysis; No body tremor. 4 = Pilorection; body stiffening; restriction of movement; paralysis; No body tremor. 5 = Pilorection; body stiffening; restriction of movement; paralysis; body tremor. Data shown is representative of three different independent experiments and ‘*n*’ represents the number of animals in each group
