# Supplementary material for: HSP60 critically regulates endogenous IL-1β production in activated microglia by stimulating NLRP3 inflammasome pathway
Source: J Neuroinflammation. 2018 Jun 9;15:177. doi: 10.1186/s12974-018-1214-5 (PMC5994257; doi:10.1186/s12974-018-1214-5)
Supplement: Supplementary file 1 — Table S1. Primers list used for quantitative real-time PCR analysis of different genes. Figure S1. Confirmation of HSP60 knockdown by vivo morpholino in mice brain. Figure S2. The overexpression of HSP60 was confirmed by Western blot. Figure S3. JEV induces inflammation in N9 murine microglial cells at MOI 2. (DOCX 1066 kb) [file 12974_2018_1214_MOESM1_ESM.docx]

| **Gene**  **Table S1: Primers list used for quantitative real time PCR analysis of different genes** | **Forward primer (5’-3’)** | **Reverse Primer (5’-3’)** |
| --- | --- | --- |
| Human HSP60 | GTG TGG CCT CTC TGT TAA CTA C | GAA CAT GCC ACC TCC CAT AC |
| Human IL-1β | CTG CGT GTT GAA AGA TGA TAA G | CCA CAT TCA GCA CAG GAC TC |
| Human GAPDH | GCA AAT TCC ATG GCA CCG T | TCG CCC CAC TTG ATT TTG G |
| Mouse HSP60 gene | GCA GAG TTC CTC AGA AGT TGG | GCA TCC AGT AAG GCA GTT CTC |
| Mouse IL-1β | TGG AAA AGC GGT TTG TCT | ATA AAT AGG TAA GTG GTT GCC |
| Mouse NLRP3 | TGC TCT TCA CTG CTA TCA AGC CCT | ACA AGC CTT TGC TCC AGA CCC TAT |
| Mouse GAPDH | ATG GCA AGT TCA AAG GCA CAG TCA | TGG GGG CAT CAG CAG AAG G |


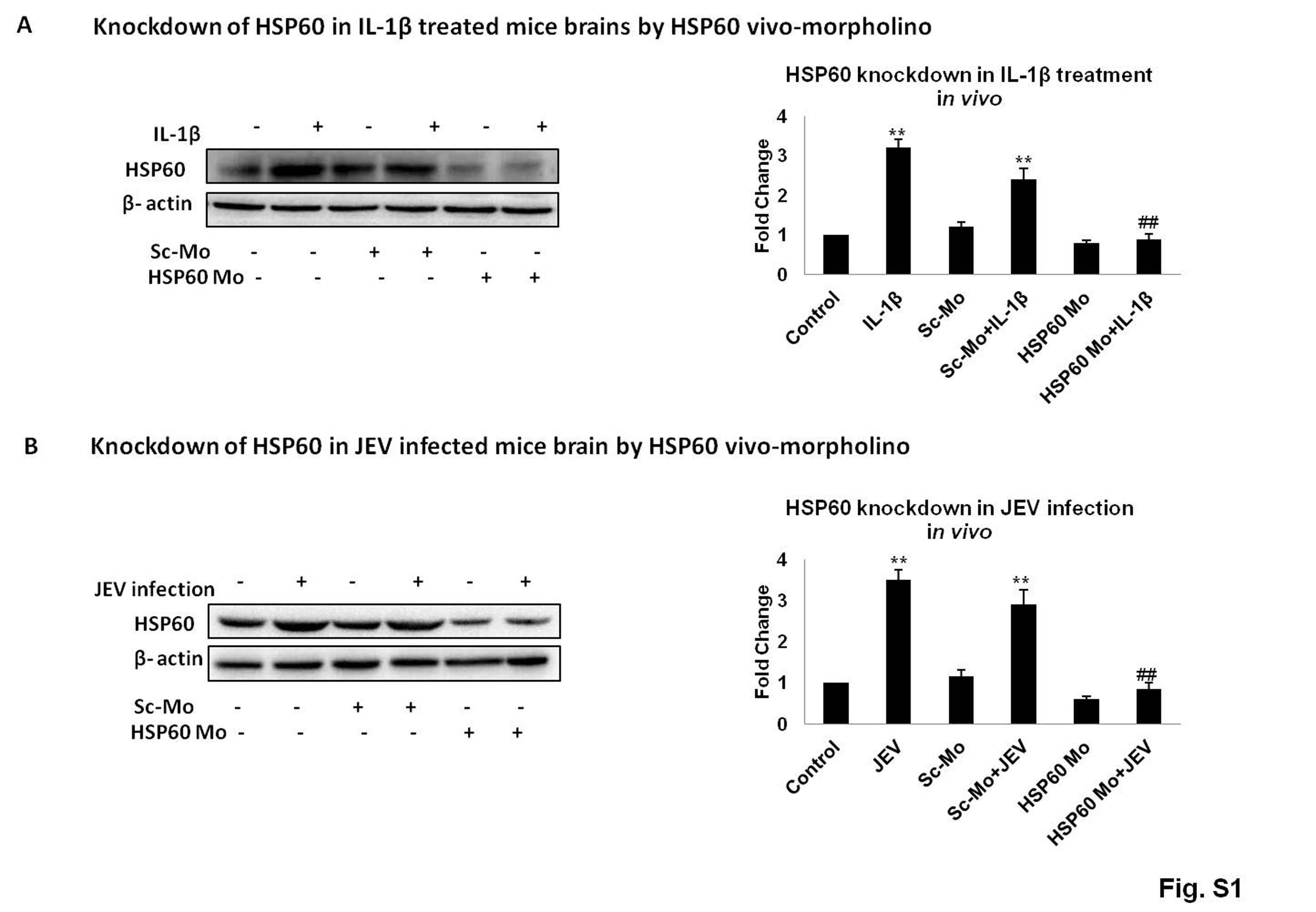


**Figure S1: Confirmation of HSP60 knockdown by vivo morpholino in mice brain. A.** HSP60 knockdown by HSP60 vivo-morpholino (HSP60-Mo) was done which significantly reduced HSP60 levels even after IL-1β treatment. **B.** Knockdown of HSP60 by HSP60- Mo in JEV infected mice brain. Bar graphs on the right panel represent the quantification of protein levels. *p<0.05, **p<0.01 in comparison to control values. ##p< 0.01 in comparison to IL-1β treatment/ JEV infection. Data represented are mean ±SD of three independent experiments.


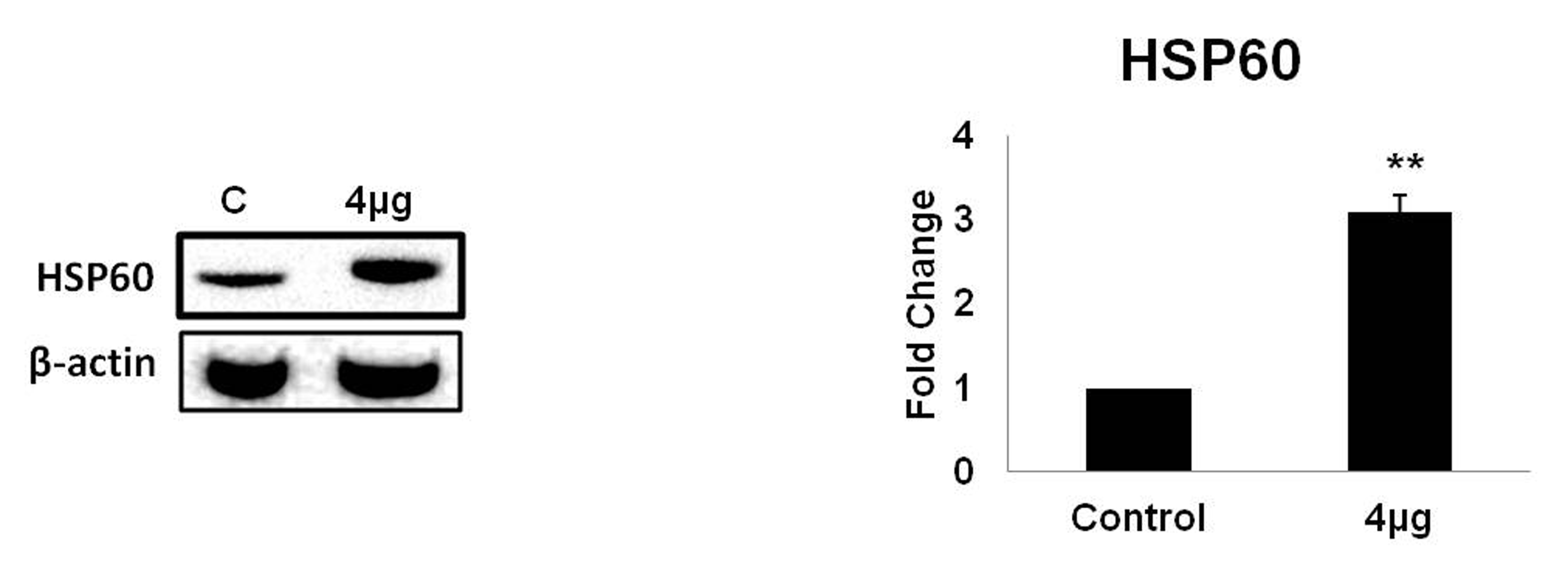


**Figure S2: The overexpression of HSP60 was confirmed by Western blot.** N9 cells transfected with 4μg mouse HSP60 cDNA plasmid clone show significant increase in HSP60 expression levels. Left panel shows the Western blot of HSP60 and β-actin, while the bar graph in the right panel shows the densitometric analysis of the Western blot. 30μg of the cytosolic protein was loaded for the Western blot. β-actin was used as a loading control. The blots are representative of three independent experiments. Data represented are mean ±SD of three independent experiments. **p<0.01 in comparison to control values.


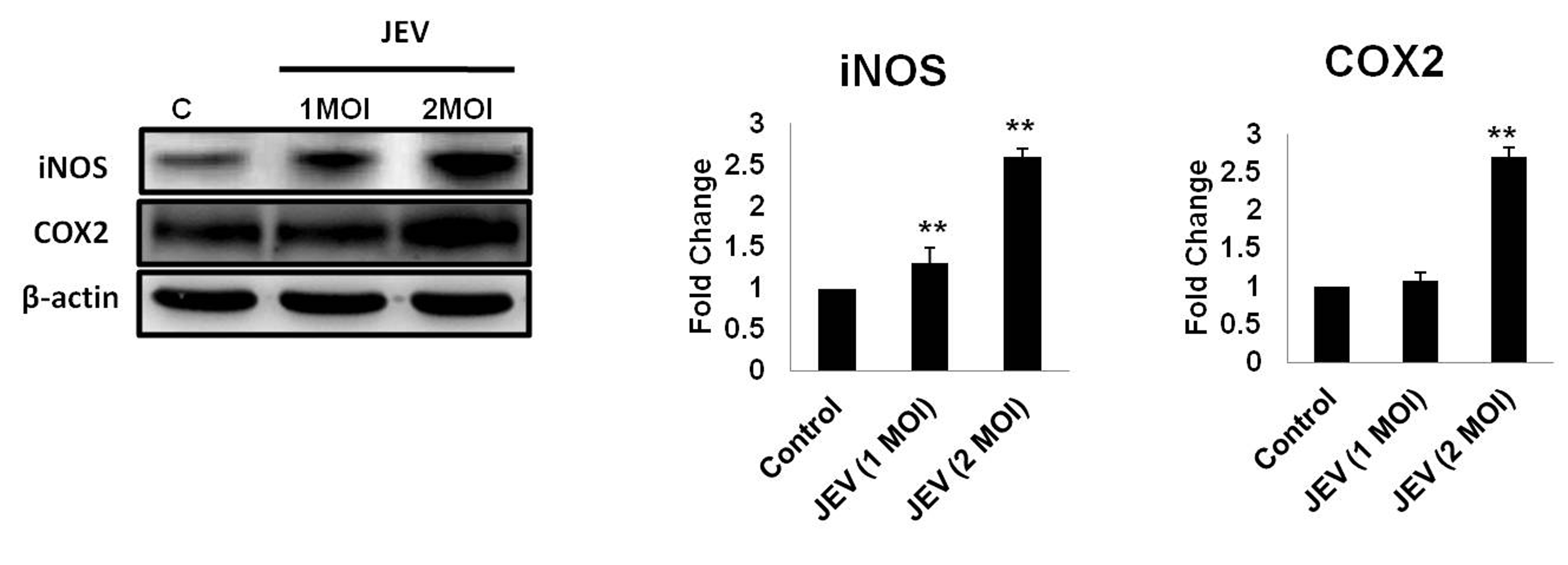


**Figure S3: JEV induces inflammation in N9 murine microglial cells at MOI 2.** Two different MOI (MOI 1 and MOI 2) were used for the infection of N9 cells. Left panel shows the Western blot analysis of iNOS and COX2 after JEV infection, while right panel shows the densitometric analysis of the Western blots. 30μg of the cytosolic protein was loaded for the Western blot and β-actin was used as a loading control. JEV significantly induces the expression of pro-inflammatory enzymes, iNOS and COX2 at MOI 2. The blots are representative of three independent experiments. Data represented are mean ±SD of three independent experiments. **p<0.01 in comparison to control values.
